# The leaf-scale mass-based photosynthetic optimization model better predicts photosynthetic acclimation than the area-based

**DOI:** 10.1093/aobpla/plae044

**Published:** 2024-08-19

**Authors:** Yuan Yu, Huixing Kang, Han Wang, Yuheng Wang, Yanhong Tang

**Affiliations:** Department of Ecology, College of Urban and Environmental Sciences, Peking University, Beijing 100871, China; Department of Ecology, College of Urban and Environmental Sciences, Peking University, Beijing 100871, China; Ministry of Education Key Laboratory for Earth System Modeling, Department of Earth System Science, Tsinghua University, Beijing 100084, China; Department of Ecology, College of Urban and Environmental Sciences, Peking University, Beijing 100871, China; Department of Ecology, College of Urban and Environmental Sciences, Peking University, Beijing 100871, China

**Keywords:** Optimization model, optimality, photosynthetic acclimation, photosynthetic capacity, the maximum Rubisco carboxylation rate

## Abstract

Leaf-scale photosynthetic optimization models can quantitatively predict photosynthetic acclimation and have become an important means of improving vegetation and land surface models. Previous models have generally been based on the optimality assumption of maximizing the net photosynthetic assimilation per unit leaf area (i.e. the area-based optimality) while overlooking other optimality assumptions such as maximizing the net photosynthetic assimilation per unit leaf dry mass (i.e. the mass-based optimality). This paper compares the predicted results of photosynthetic acclimation to different environmental conditions between the area-based optimality and the mass-based optimality models. The predictions are then verified using the observational data from the literatures. The mass-based optimality model better predicted photosynthetic acclimation to growth light intensity, air temperature and CO_2_ concentration, and captured more variability in photosynthetic traits than the area-based optimality models. The findings suggest that the mass-based optimality approach may be a promising strategy for improving the predictive power and accuracy of optimization models, which have been widely used in various studies related to plant carbon issues.

## Teaser

To address the debate on whether photosynthetic optimization models should assume that leaves optimize their net carbon assimilation per unit leaf area or per unit leaf dry mass, we developed models based on the area-based optimality and the mass-based optimality. We then compared the model predictions with the data from the literature. We present the first evidence that the mass-based optimality model outperforms the area-based optimality models in predicting photosynthetic acclimation to growth light intensity, air temperature and CO_2_ concentration. The mass-based optimality model captures more variability in leaf photosynthetic traits than the area-based optimality models. The results suggest that the mass-based optimality model may be a promising method for improving predictions of photosynthetic traits in different environments and under future climate change.

## Introduction

Leaves adjust their photosynthetic traits by changing anatomical structure and biochemical components in response to growth conditions such as light intensity, air temperature and CO_2_ concentration, which is commonly known as photosynthetic acclimation (Armond *et al.* 1978; Givnish 1988). A common case is the variation in leaf traits within species between sun-exposed and shaded leaves. Leaves grown in higher light intensity generally have more layers or larger volumes of leaf mesophyll cells. This allows for more photosynthetic substances to be accommodated, resulting in an increase in leaf dry mass per unit leaf area (LMA), maximum carboxylation rate of Rubisco per unit leaf area at 25 °C ($V_{c\;max,25}$) and light-saturated photosynthetic rate per unit leaf area ($A_{max}$) (Givnish 1988; Niinemets and Tenhunen 1997; Niinemets *et al.* 1999, 2006; Frak 2002; Niinemets 2007; Tong and Ng 2008; Buckley *et al.* 2013; Mathur *et al.* 2018). Besides, leaves grown under strong light generally age faster and have a shorter lifespan (T) (Vincent 2006; Xu *et al.* 2017). Accounting for photosynthetic acclimation can improve the accuracy of the existing ecosystem models in predicting gross primary productivity (Haxeltine and Prentice 1996; Wang *et al.* 2017; Franklin *et al.* 2020; Luo and Keenan 2020; Harrison *et al.* 2021). Therefore, it is crucial to comprehend and predict photosynthetic acclimation.

The leaf-scale optimality hypothesis suggests that leaves adjust their photosynthetic traits through photosynthetic acclimation to maximize leaf net photosynthetic carbon assimilation, which helps plants increase their fitness and then survive in natural selection (Bjorkman *et al.* 1972; Osmond *et al.* 1980; Givnish 1988; Franklin *et al.* 2020; Harrison *et al.* 2021). Leaf-scale photosynthetic optimization models based on this hypothesis set leaf net carbon assimilation as the variable to be maximized (i.e. fitness proxy) and represent it as a function of leaf photosynthetic traits (e.g. nitrogen content or $V_{c\;max,25}$) that are the variables to be optimized (Franklin *et al.* 2020). These models quantitatively predict the acclimation of photosynthetic traits in various growth environments (Field 1983; Farquhar 1989; Haxeltine and Prentice 1996; Niinemets 2012; Buckley *et al.* 2013; Retkute *et al.* 2015; Wang *et al.* 2017; Franklin *et al.* 2020; Harrison *et al.* 2021; Joshi *et al.* 2022).

However, there is controversy regarding whether the leaf-scale optimality hypothesis should target the net carbon assimilation per unit leaf area or per unit leaf dry mass (Bjorkman *et al.* 1972; Givnish 1988). Through a light control experiment on *Atriplex triangularis*, Bjorkman *et al.* (1972) found that respiration rate per unit leaf area (R) and $A_{max}$ increase with increasing growth light intensity, which suggests that leaves acclimated to a particular growth light may have a higher net photosynthetic carbon assimilation than those from other growth light. Therefore, it has been proposed that photosynthetic acclimation may favour leaves that achieve the highest net photosynthetic carbon assimilation per unit leaf area in their growth environments (Bjorkman *et al.* 1972), known as the area-based optimality. However, Givnish (1988) reanalysed Bjorkman’s (1972) data and found that the daily net photosynthetic carbon assimilation per unit leaf area of the leaves acclimated to low growth light intensity is 29% lower in low light intensity than that of the leaves acclimated to high growth light intensity (Givnish 1988). This finding contradicts the idea of the area-based optimality. However, when net carbon assimilation is expressed on a unit leaf dry mass basis, the optimality re-emerges. This means that leaves acclimated to a specific growth light intensity have a higher daily net photosynthetic carbon assimilation per unit leaf dry mass in this light intensity than those acclimated to other growth light intensities (Givnish 1988). Therefore, Givnish (1988) proposed that leaves may maximize the net photosynthetic carbon assimilation per unit leaf dry mass (i.e. the mass-based optimality) through photosynthetic acclimation, rather than that per unit leaf area as suggested by Bjorkman *et al.* (1972) in their area-based optimality.

Both the area-based optimality and the mass-based optimality assume that photosynthetic traits are subject to natural selection to increase fitness (Osmond *et al.* 1980; Givnish 1988; Mathur *et al.* 2018; Franklin *et al.* 2020). The implicit assumptions of them are that the targets which leaves need to optimize through photosynthetic acclimation should contribute to plant growth (Franklin *et al.* 2020; Harrison *et al.* 2021). The optimal target of the area-based optimality and that of the mass-based optimality are the net photosynthetic carbon assimilation per unit leaf area and that per unit leaf dry mass, respectively. In fact, the net photosynthetic carbon assimilation per unit leaf dry mass has a closer relationship with the growth of leaves or individuals compared to that per unit leaf area (Poorter *et al.* 1990; Garnier 1991). Both the relative leaf area expansion rate and the relative growth rate of an individual plant are closely correlated with the net photosynthetic carbon assimilation rate per leaf dry mass. However, they are weakly correlated with that per unit leaf area (Poorter *et al.* 1990; Garnier 1991; Lambers and Poorter 1992; Bazzaz and Grace 1997; Lambers *et al.* 2008). These results support the implicit assumption of the mass-based optimality but undermine that of the area-based optimality. This casts further doubt on the correctness of the area-based optimality.

Although the above results support the mass-based optimality, previous leaf-scale photosynthetic optimization models have generally adopted the area-based optimality without any reason or evidence against the mass-based optimality (Farquhar 1989; Haxeltine and Prentice 1996; Niinemets 2012; Buckley *et al.* 2013; Retkute *et al.* 2015; Wang *et al.* 2017; Smith *et al.* 2019; Franklin *et al.* 2020; Luo and Keenan 2020; Harrison *et al.* 2021; Joshi *et al.* 2022; Deans *et al.* 2020). These models predict that $V_{c\;max,25}$, $A_{max}$, and A are proportional or approximately proportional to growth light intensity, and that $A_{max}$ and A continue to decrease with increasing growth air temperature (Wang *et al.* 2017; Smith *et al.* 2019; Luo and Keenan 2020; Joshi *et al.* 2022). However, these predictions are inconsistent with the observations. In fact, the increase in $V_{c\;max,25}$, $A_{max}$ and A slows with the increase in growth light intensity (Niinemets 2012; Buckley *et al.* 2013; Niinemets *et al.* 2015; Poorter *et al.* 2019). $A_{max}$ and A increase first and then decreases with increasing growth air temperature and 20–30 °C may be the optimal temperature of leaf photosynthesis for most species (Scafaro *et al.* 2017; Cai *et al.* 2020). Efforts have been made to address these issues by incorporating more physiological mechanisms into the models, yet they have not been fully resolved (Niinemets 2012; Buckley *et al.* 2013; Joshi *et al.* 2022). We speculated that these issues may be rooted in the area-based optimality and that the mass-based optimality may help improve leaf-scale photosynthetic optimization models and address these problems. However, few studies have explored the potential application of the mass-based optimality in leaf-scale photosynthetic optimization models.

This study is to address whether leaf-scale photosynthetic optimization models should assume that leaves optimize their net carbon assimilation per unit leaf area (i.e. the area-based optimality) or that per unit leaf dry mass (i.e. the mass-based optimality). We developed models based on the area-based optimality and the mass-based optimality, respectively. We then compared the predicted results with the observed data from the literature to assess the impact of the two optimality on the leaf-scale optimization models.

## Method

### The model frameworks of the two optimality criteria

The photosynthetic acclimation discussed in this article referred to the intraspecific variation of photosynthetic traits between different leaves grown in different environments rather than the temporal variation of photosynthetic traits in one leaf caused by the temporal variation of growth environments (Athanasiou *et al.* 2010). To simplify models and facilitate model solution, we ignored the daily and the annual changes in environment factors and assumed that the environments is the same every day. The value of environmental factors was set as the average value, for example, the growth light intensity during daytime was set as the average daytime light intensity. This article tried to predict five main leaf traits, that is, the maximum carboxylation rate of Rubisco per unit leaf area at 25 °C ($V_{c\;max,25}$), the light-saturated photosynthetic rate per unit leaf area ($A_{max}$), the photosynthetic rate per unit leaf area (A), the leaf dry mass per unit leaf area (LMA) and leaf lifespan (T), through photosynthetic optimization models based on the area-based optimality and the mass-based optimality.

The area-based optimality and the mass-based optimality assumes, respectively, that a leaf maximizes the net photosynthetic carbon gain rate per unit leaf area per unit time ($P_{a},\;\;\mu \;mol\;\;\;C\;\;\;m^{-2}\;\;\;day^{-1}$) or per unit leaf dry mass per unit time ($P_{m},\;\;\mu \;mol\;\;\;C\;\;\;g^{-1}\;\;\;day^{-1}$), over the entire lifespan:


$$\begin{array}{l} \begin{array}{l} \max(P_{a})=\max((G-C_{c})/T),\; \end{array} \end{array}$$ (1)

$$\begin{array}{l} \begin{array}{l} \max(P_{m})=\max((G-C_{c})/T/LMA),\; \end{array} \end{array}$$ (2)

where G$\left(\;\mu \;mol\;\;\;C\;\;\;m^{-2}\right)$ is the total net carbon gain for the whole lifespan, $C_{c}$ ($\;\mu \;mol\;\;\;C\;\;\;m^{-2}$) is the total construction cost per unit leaf area, T (day) is the duration (days) for the entire leaf lifespan and LMA ($g\;\;\;m^{-2}$) is the leaf dry mass per unit leaf area. There is a close relationship among G, $C_{c}$ and LMA because they are all the functions of $V_{c\;max,25}$. The following is their modelling process.

### Modelling G

The total net carbon gain for the entire leaf lifespan (G,$\;\mu \;mol\;\;\;C\;\;\;m^{-2}$) is determined by photosynthesis, respiration and leaf lifespan (Kikuzawa 1991):


$$\begin{array}{l} \begin{array}{l} A_{n\;day}(t)=A_{n\;day}(0)\left(1-\frac{t}{LL_{p}}\right),\; \end{array} \end{array}$$ (3)

$$\begin{array}{l} \begin{array}{l} G=\overset{T}{\underset{0}{\int }}A_{n\;day}(t)dt=A_{n\;day}(0)\left(T-\frac{T^{2}}{2LL_{p}}\right),\; \end{array} \end{array}$$ (4)

where t (day) is the number of days since the leaf was born, T (day) is the number of days for the entire leaf lifespan, $A_{n\;day}\left(t\right)$ and $A_{n\;day}\left(0\right)$ ($\;\mu \;mol\;\;\;C\;\;\;m^{-2}\;\;\;day^{-1}$) are the daily net carbon assimilation per unit leaf area at the leaf age of t days and 0 day, respectively, and $LL_{p}$ (day) is the duration when $A_{n\;day}\left(t\right)$ decreases to 0. According to Kikuzawa and Lechowicz (2006) and Xu *et al.* (2017), $LL_{p}$ is negatively related to the daily net carbon assimilation per unit leaf dry mass [see **Supporting Information—**Supplementary Material 4, $R^{2}=0.75$]:


$$\begin{array}{l} \begin{array}{l} LL_{p}=m{\left(\frac{A_{n\;day}\left(0\right)}{LMA}\right)}^{n},\; \end{array} \end{array}$$ (5)

where m is set as $2.37\times 10^{7}$ and n is set as −4/3 (the fitting results of Supporting Information—Supplementary Material 4). The specific physiological mechanisms of this relationship are still under debate (Xu *et al.* 2017).

Assuming that the proportion of daytime to the entire day is p, $A_{n\;day}\left(0\right)$ is:


$$\begin{array}{l} \begin{array}{l} A_{n\;day}\left(0\right)=\left(\;p\times A-R\right)\times 24\times 3600,\; \end{array} \end{array}$$ (6)

where A and R ($\;\mu \;mol\;\;\;C\;\;\;m^{-2}\;\;\;s^{-1}$) are the photosynthetic and the respiration rate per unit leaf area at the leaf of 0 day, respectively.

According to the right-angle hyperbola formula of the light response curve, A is:


$$\begin{array}{l} \begin{array}{l} A=\frac{\alpha IA_{max}}{\alpha I+A_{max}},\; \end{array} \end{array}$$ (7)

where $A_{max}$ ($\;\mu \;mol\;\;\;m^{-2}s^{-1}$) is the light-saturated photosynthetic rate, α is the apparent quantum yield, and I is the photosynthetic photon flux density ($\;\mu \;mol\;\;\;m^{-2}s^{-1}$). According to the FvCB model (Farquhar *et al.* 1980), considering the limitation by Rubisco and RuBP regeneration, $A_{max}$ and α can be derived [see Supporting Information—Supplementary Material 5]:


$$\begin{array}{l} \begin{array}{l} A_{max}=\min\left(\frac{V_{c\;max,25}f_{V_{c\;max}}(C_{ch}-{\;\Gamma \;}^{*})}{C_{ch}+K},\frac{J_{max,25}f_{J_{max}}(C_{ch}-{\;\Gamma \;}^{*})}{4C_{ch}+8{\;\Gamma \;}^{*}}\right),\; \end{array} \end{array}$$ (8)

$$\begin{array}{l} \begin{array}{l} J_{max,25}=r_{J,V}V_{c\;max,25},\; \end{array} \end{array}$$ (9)

$$\begin{array}{l} \begin{array}{l} \alpha =\frac{abs\cdot R_{PSII}\cdot {\;\Phi \;}_{PSII}\cdot (1-l)}{4}\cdot \frac{C_{ch}-\Gamma^{*}}{C_{ch}+2\Gamma^{*}},\; \end{array} \end{array}$$ (10)

where $V_{c\;max,25}$ ($\;\mu \;mol\;\;\;CO_{2}\;m^{-2}s^{-1}$) is the maximum carboxylation rate of Rubisco at 25 °C, $f_{V_{c\;max}}$ and $f_{J_{max}}$ are the leaf temperature-dependent function of $V_{c\;max}$ and the maximum potential electron transfer rate ($J_{max}$, $\;\mu \;mol\;\;\;m^{-2}s^{-1}$), respectively, $r_{J,V}$ is the ratio of the maximum potential electron transfer rate at 25 °C ($J_{max,25}$, $\;\mu \;mol\;\;\;m^{-2}s^{-1}$) to $V_{c\;max,25}$, $C_{ch}$ (ppm) is the chloroplastic CO_2_ concentration, ${\;\Gamma \;}^{*}$ (ppm) is the photosynthetic CO_2_ compensation point without dark respiration, K (ppm) is the Michaelis–Menten constants, abs is the absorbance of photosynthetic photon flux density by leaves (set as 0.84), and $R_{PSII}$ is the proportion of captured light energy allocated to photosystem II in low light (set as 0.5) in low light and l is the spectral correction factor of white light (set as 0.15) and ${\;\Phi \;}_{PSII}$ is the maximum quantum yield of photosystem II (set as 0.8). $f_{V_{c\;max}}$, $f_{J_{max}}$, $r_{J,V}$, ${\;\Gamma \;}^{*}$ and K are influenced by leaf temperature ($T_{leaf}$) and are the functions of $T_{leaf}$ [see Supporting Information—Supplementary Material 5]. $T_{leaf}$ was assumed to be equal to growth air temperature ($T_{air}$, °C). $C_{ch}$ is the function of leaf vapour pressure deficit (VPD, $kP_{a}$) and atmosphere CO_2_ concentration ($C_{a}$, ppm) (Medlyn *et al.* 2011) [see Supporting Information—Supplementary Material 5]:


$$\begin{array}{l} \begin{array}{l} C_{ch}=\frac{g_{1}-0.89\sqrt{VPD}}{g_{1}+\sqrt{VPD}}C_{a},\; \end{array} \end{array}$$ (11)

where $g_{1}$ ($k{P_{a}}^{0.5}$) is a parameter that is different among different species. Here, $g_{1}$ is set as 3.6 $k{P_{a}}^{0.5}$, which is the average value of different species (Medlyn *et al.* 2011).

Leaf respiration rate per unit leaf area ($R,\;\mu mol\;\;\;C\;\;\;m^{-2}\;\;\;s^{-1}$) is assumed to be proportional to $V_{c\;max,25}\;$(Wang *et al.* 2020) and influenced by leaf temperature ($T_{leaf}$, K) (Heskel *et al.* 2016):


$$\begin{array}{l} \begin{array}{l} R=f_{R}(T_{leaf})\beta_{R}V_{c\;max,25},\; \end{array} \end{array}$$ (12)

where $f_{R}$ is the leaf temperature-dependent function of R and $\beta_{R}$ is the ratio coefficient, which can be set as 0.015 based on previous measurement results (Wang *et al.* 2020).

### Modelling LMA and $C_{c}$

Leaf dry mass per unit leaf area (LMA, $g\;\;\;m^{-2}$) relates to $V_{c\;max,25}$, and hence relates to leaf construction cost per unit leaf area ($C_{c}$, $\;\mu \;mol\;\;\;C\;\;\;m^{-2}$). The rationale underpinning such a relation is that with the increase in the layers and volumes of leaf mesophyll cells, unit leaf area can accommodate more photosynthetic protein (e.g. Rubisco), which leads to an increase in $V_{c\;max,25}$ and then increase $A_{max}$ and A (Givnish 1988; Poorter *et al.* 2019; Mizokami *et al.* 2022). The increase in leaf mesophyll cells and photosynthetic protein per unit leaf area would lead to an increase in leaf dry mass per unit leaf area (LMA) (Poorter *et al.* 2009; Villar *et al.* 2013; John *et al.* 2017; Mizokami *et al.* 2022), and would also cost more carbohydrate to provide energy to construct leaves (Bazzaz and Grace 1997; Barthod and Epron 2005; Poorter *et al.* 2006; Villar *et al.* 2006; Feng *et al.* 2008), which then increase $C_{c}$. Previous data show that $V_{c\;max,25}$ linearly contributes to LMA (Equation (13) and Supporting Information—Supplementary Material 2):


$$\begin{array}{l} \begin{array}{l} LMA=kV_{c\;max,25}+b\;(k>0,\;b>0),\; \end{array} \end{array}$$ (13)

where k represents the increased LMA per unit increased, and $V_{c\;max,25}$ and b represent the LMA when $V_{c\;max,25}$ is 0. The evidence regarding Equation (13) can be found in Supporting Information—Supplementary Material 2. k and b can be obtained by fitting the paired LMA and $V_{c\;max,25}$ using Equation (13) [see Supporting Information—Supplementary Material 2].

k and b vary among species but can be regarded as constants within species [Supporting Information—Supplementary Material 2; e.g., Ellsworth and Reich 1993; Niinemets and Valladares 2004; Turnbull *et al.* 2007]. Although many studies have observed the intraspecific linear relationship between LMA and $V_{c\;max,25}$, few researches study the reasons for the interspecific variation in k and b. According to the anatomical and compositional basis of LMA (John *et al.* 2017), we speculated that k and b may relate to leaf anatomical structure. k maybe larger theoretically if the unit dry mass of mesophyll cells can accommodate more Rubisco (e.g. the same volume of mesophyll cells accommodates more chloroplasts and then increase the content of Rubisco, or mesophyll cells have thinner cell wall thickness to decrease the mass of mesophyll cells) (Shipley *et al.* 2006; John *et al.* 2017; Onoda *et al.* 2017). b may relate to non-photosynthetic tissues (e.g. cuticle and epidermis) that have little effects on $V_{c\;max,25}$ (John *et al.* 2017).

$C_{c}$ is generally proportional to LMA. Constructing 1 g of dried leaves requires an average of 1.5 g glucose (Bazzaz and Grace 1997; Barthod and Epron 2005; Poorter *et al.* 2006; Villar *et al.* 2006; Feng *et al.* 2008):


$$\begin{array}{l} \begin{array}{l} C_{c}=\beta_{c}LMA,\; \end{array} \end{array}$$ (14)

where $\beta_{c}$ is the coefficient between $C_{c}$ and LMA, which is set as 50 000 $\;\mu \;mol\;\;\;C\;\;\;g^{-1}$ (equivalent to 1.5 g glucose/g dried leaf).

### The solution of the photosynthetic optimization models

According to Equation (1)–Equation (14), we calculated the numerical solutions of the $V_{c\;max,25}$ and the T that maximize $P_{a}$ (i.e. area-based optimality, hereafter referred to it as A-model) and maximize $P_{m}$ (i.e. mass-based optimality, hereafter referred to it as M-model), respectively. Then, combining Equation (7), Equation (8) and Equation (13), $A_{max}$, A and LMA can be calculated.

Obtaining accurate analytical solutions for the two models is difficult. We found the approximate analytical solutions of the two models. Through the approximate analytical solutions, the two models’ predictions of photosynthetic acclimation to different environments can be easily and intuitively reflected. To simplify writing, we set $f_{V}$ as:


$$\begin{array}{l} \begin{array}{l} f_{V}=\min\left(\frac{f_{V_{c\;max}}(C_{ch}-{\;\Gamma \;}^{*})}{C_{ch}+K},\frac{r_{J,V}f_{J_{max}}(C_{ch}-{\;\Gamma \;}^{*})}{4C_{ch}+8{\;\Gamma \;}^{*}}\right).\; \end{array} \end{array}$$ (15)

So, the A-model and the M-model predicts that $V_{c\;max,25}$ are:


$$\begin{array}{l} \begin{array}{l} {V_{c\;max,25}}_{A}=\frac{\alpha I}{f_{V}}\left(\sqrt{\frac{pf_{V}}{\beta_{R}f_{R}}}-1\right),\; \end{array} \end{array}$$ (16)

$$\begin{array}{l} \begin{array}{l} V_{c\;max,25\;M}=\frac{\sqrt{\frac{\alpha I}{f_{V}}\frac{b}{k}(1-\beta_{R}f_{R}/pf_{V})+{\left(b/k\right)}^{2}\beta_{R}f_{R}/pf_{V}}-b/k(\beta_{R}f_{R}/pf_{V})}{1+\beta_{R}f_{R}/p(b/\alpha Ik)}.\; \end{array} \end{array}$$ (17)

The subscript ‘A’ and ‘M’ in the lower right corners of $V_{c\;max,25}$ indicate that they are predicted by the A-model and the M-model, respectively. Both models predict that T is:


$$\begin{array}{l} \begin{array}{l} T=\sqrt{2m\beta_{c}{\left(\frac{86\;400\left(\;pA-R\right)}{LMA}\right)}^{n-1}}\; \end{array}, \end{array}$$ (18)

where A and R are the photosynthetic rate and respiration rate per unit leaf area predicted by the A-model or the M-model, respectively.

### Model verification

To compare the differences in prediction between our model with previous models, the widely used P-model (Wang *et al.* 2017; Smith *et al.* 2019; Joshi *et al.* 2022), which is also a typical area-based optimality model, was also compared with the A-model and the M-model. The equation of the P-model can be found in Supporting Information—Supplementary Material 5 (Wang *et al.* 2017; Smith *et al.* 2019; Joshi *et al.* 2022).

(1) Verifying the predicted trends of photosynthetic acclimation to various environmental factors semi quantitatively

The three optimality models were compared for predicting photosynthetic acclimation to light intensity (I), air temperature ($T_{air}$) and air CO_2_ concentration ($C_{a}$). To eliminate the impacts of species and highlight the impacts of environmental factors, this article referred to the method used by Poorter *et al.* for meta-analysis (Poorter *et al.* 2019, 2022) and mainly compared the predicted trends of photosynthetic acclimation. The predicted trends were presented in the form of relative predicted values, that is, the ratio of predicted values to the predicted values of ‘standard’ environment (I is $500\;\;\mu \;mol\;\;\;m^{-2}\;s^{-1}$, $T_{leaf}$ is 298.15 K, $C_{a}$ is 450 ppm and O_2_ is 210 $mmol\;\;\;mol^{-1}$). The predicted results of the three models were semi-quantitatively verified using the observational datasets from the literatures (Scafaro *et al.* 2017; Poorter *et al.* 2019, 2022; Cai *et al.* 2020). Poorter *et al.* (2019 and 2022) provided the fitted functions of the acclimation to I and $C_{a}$. Scafaro *et al.* (2017) and Cai *et al.* (2020) provided the fitted functions of the air temperature acclimation. However, data of leaf lifespan are not available from these studies. Thus, we did not verify the predicted trends of leaf lifespan here.

(2) Verifying photosynthetic acclimation to growth light intensity quantitatively

We compiled a dataset to verify photosynthetic acclimation to growth light intensity quantitatively [see Supporting Information—Supplementary Material 3].

First, the analytical solution of A-model (Equation (16)), M-model (Equation (17)) and P-model [see Supporting Information—Supplementary Material 5] all predict the power-law relationship between $V_{c\;max,25}$ and growth light intensity. If the relative photosynthetic capacities per unit leaf area (RP) and the relative growth light intensity (RI) were defined as follows:


$$\begin{array}{l} \begin{array}{l} RP=\frac{V_{c\;max,25}}{V_{c\max,25I_{max}}},\; \end{array} \end{array}$$ (19)

$$\begin{array}{l} \begin{array}{l} RI=\frac{I}{I_{max}},\; \end{array} \end{array}$$ (20)

where I is the growth light intensity, $V_{c\;max,25}$ is the photosynthetic traits of leaves grow in I. $I_{max}$ is the maximum value of light intensity in the growth light intensity gradient and $V_{c\;max,25\;I_{max}}$ is the $V_{c\;max,25}$when I is $I_{max}$. A-model and P-model both predicted that $V_{c\;max,25}$ is proportional to 1 power of growth light intensity:


$$\begin{array}{l} \begin{array}{l} RP=RI.\; \end{array} \end{array}$$ (21)

while the M-model predicted that $V_{c\;max,25}$ is approximately proportional to 0.5 power of growth light intensity:


$$\begin{array}{l} \begin{array}{l} RP=\sqrt{RI}.\; \end{array} \end{array}$$ (22)

Second, we quantitatively predicted $V_{c\;max,25}$ and T of different species in different growth light intensities using the A-model, the M-model and the P-model, respectively. The prediction results were then verified using our dataset.

## Results

### The M-model predicts a lower $V_{c\;max,25}$ than the A-model

**Figure 1.**
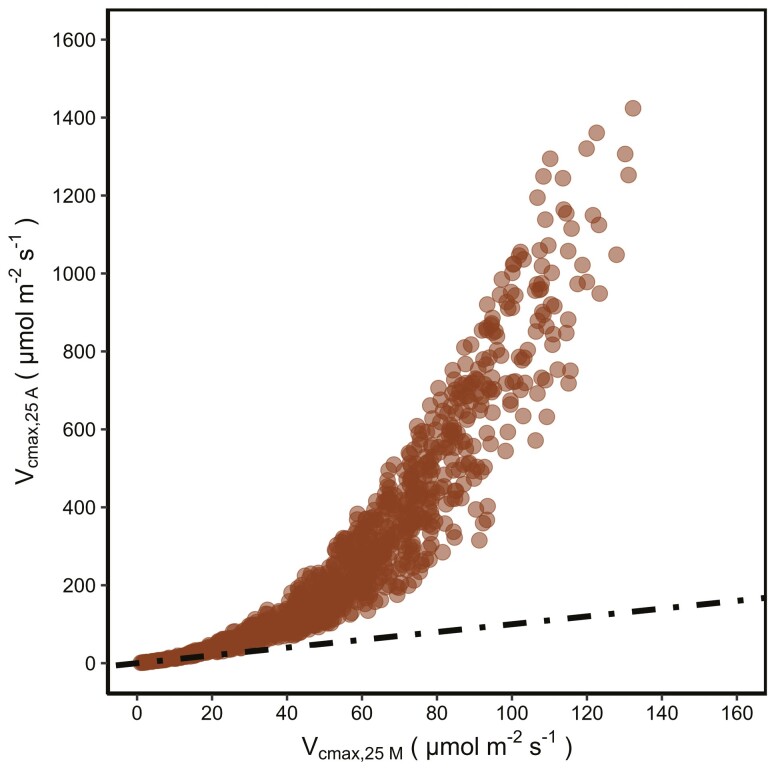
The comparison of the $V_{c\;max,25}$ predicted by the A-model and the M-model: randomly inputting values of I, $C_{a}$ and $T_{leaf}$ into the A-model and the M-model, and then comparing their predicted $V_{c\;max,25}$. The ranges of values for $I\;(\;\mu \;mol\;\;\;m^{-2}\;s^{-1})$, $C_{a}\;(ppm)$ and $T_{leaf}(K)$ were [1,1000], [200, 1000] and [273.15, 313.15], respectively. $V_{c\;max,25\;A}$ and $V_{c\;max,25\;M}$ are the $V_{c\;max,25}$ predicted by the A-model and the M-model, respectively. The dotted black line is a 1:1 line.

Fig.1 shows that the A-model and the M-model would produce different predicted $V_{c\;max,25}$ when environmental parameters are the same. The $V_{c\;max,25}$ predicted by the M-model is generally lower than that of the A-model (Fig. 1).

### Predicting the trends of photosynthetic acclimation to various environmental factors

**Figure 2.**
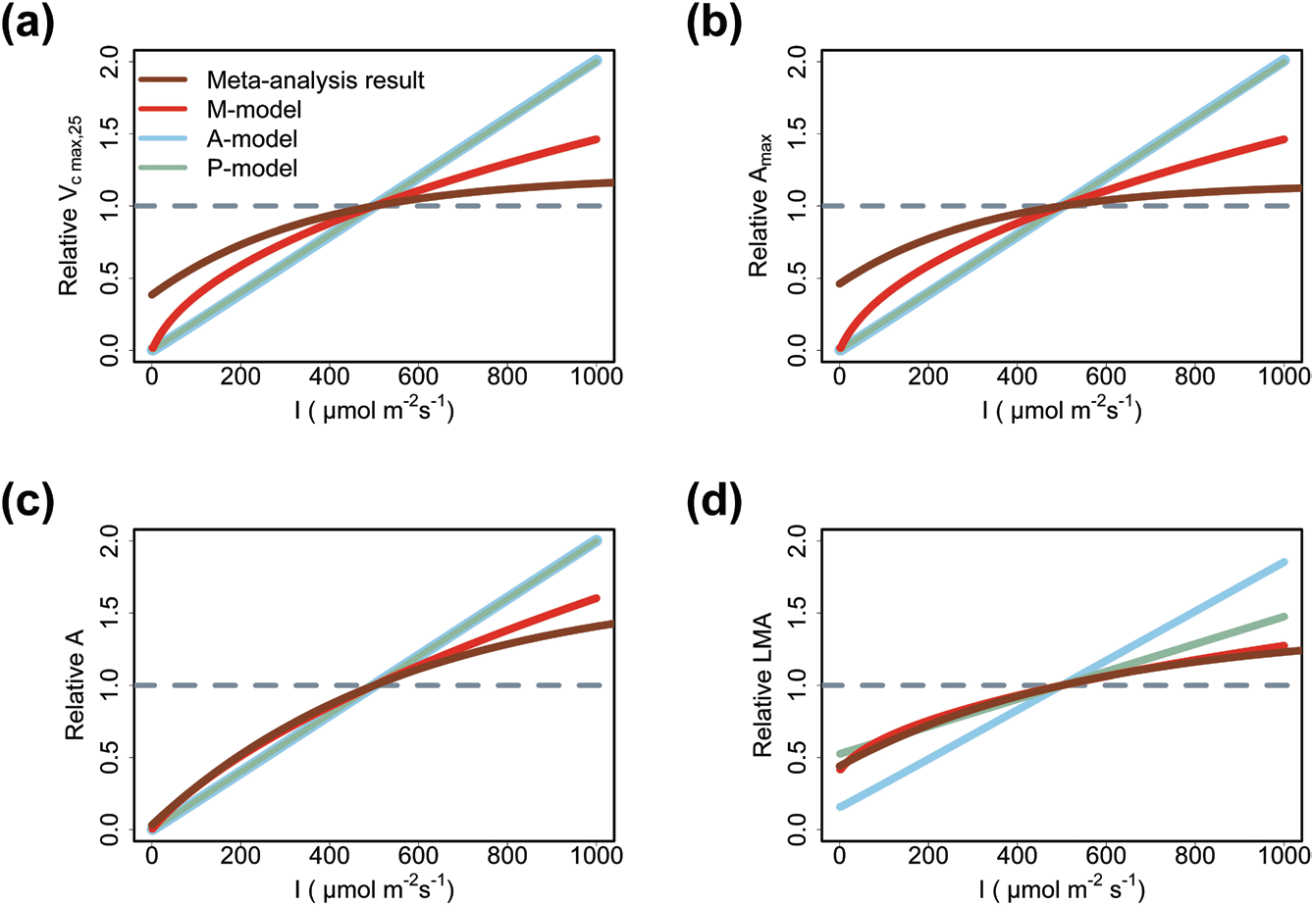
Predicted $V_{c\;max,25}$, $A_{max}$, A and LMA in relation to growth light intensity (I): predicting the acclimation trends of (A) $V_{c\;max,25}$, (B) $A_{max}$, (C) A and (D) LMA to growth light intensity (I). The prediction results of the A-model and the P-model overlap because they all predicted that $V_{c\;\;\;max,25}$, $A_{max}$ and A are proportional to I. The meta-analysis results come from Poorter *et al.* (2019).

For growth light intensity (I), the P-model and the A-model both predict a linear increase in $V_{c\;max,25}$, $A_{max}$, A and LMA with increasing I (Fig. 2). Meanwhile, the M-model predicts that the increase in $V_{c\;max,25}$, $A_{max}$, A and LMA would gradually slow (Fig. 2). The meta-analysis results (Poorter *et al.* 2019) showed that with the increase in growth light intensity, the change trends of $V_{c\;max,25}$, $A_{max}$, A and LMA becomes more similar to the gradually slowing nonlinear growth predicted by the M-model rather than the linear growth predicted by the A-model and the P-model.

**Figure 3.**
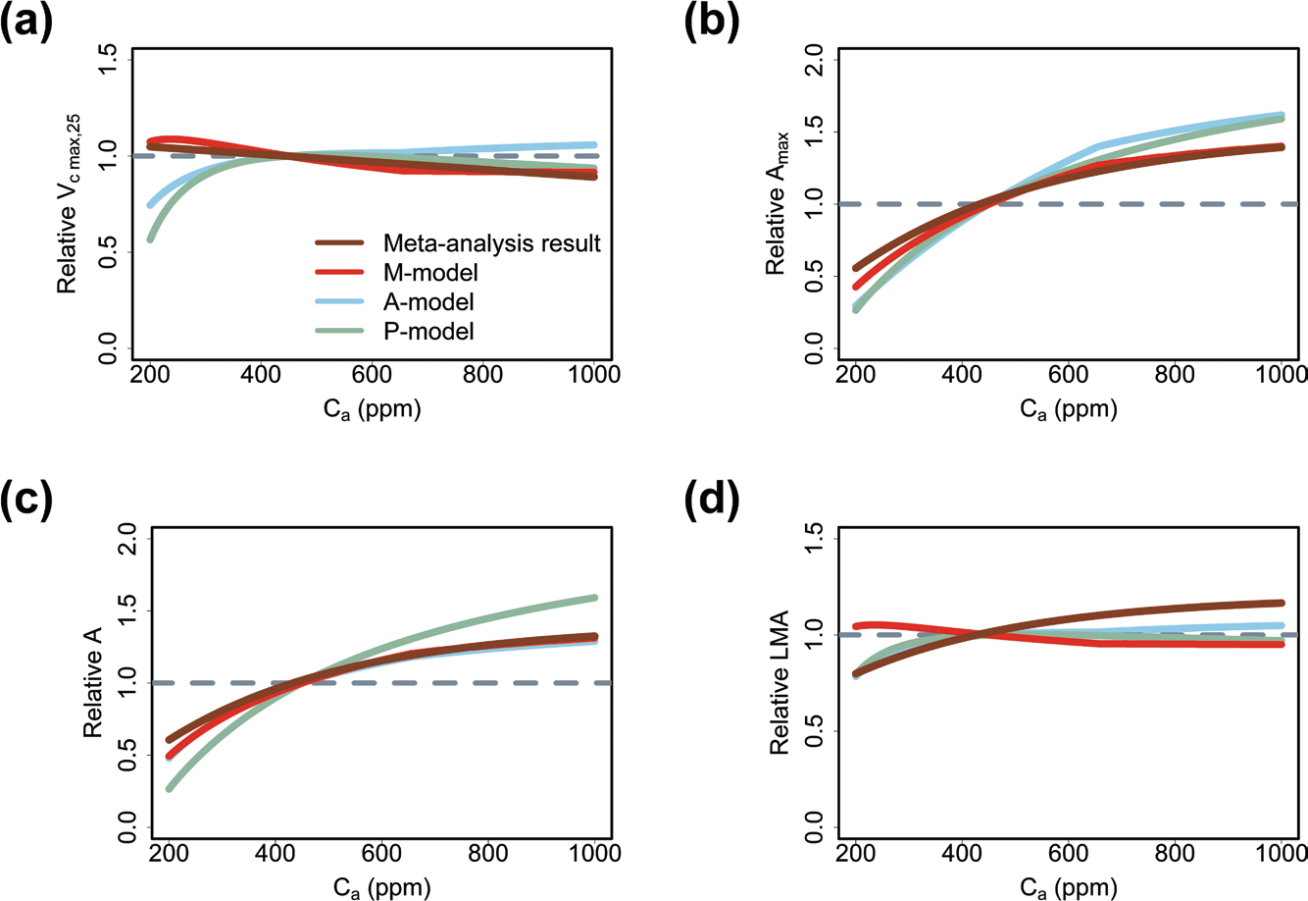
Predicted $V_{c\;\max,25}$, $A_{\max}$, A and LMA in relation to growth atmospheric CO_2_ concentration ($C_{a}$): predicting the acclimation trends of (A) $V_{c\;max,25}$, (B) $A_{max}$, (C) A and (D) LMA to growth atmospheric CO_2_ concentration ($C_{a}$). The meta-analysis results come from Poorter *et al.* (2022).

For growth atmospheric CO_2_ concentration ($C_{a}$), the A-model predicts that $V_{c\;max,25}$ continuously increases with the increase in $C_{a}$. The P-model predicts that $V_{c\;max,25}$ increases first and then decreases with increasing $C_{a}$ (Fig. 3A). However, the meta-analysis results (Poorter *et al.* 2022) show that $V_{c\;max,25}$ decreases continuously with increasing $C_{a}$, which is consistent with the predicted results of the M-model (Fig. 3A). The three models all predict that $A_{max}$ and A increase with increasing $C_{a}$ (Fig. 3B and C). The prediction of the increase in $A_{max}$ and A by the M-model is most consistent with the meta-analysis results (Poorter *et al.* 2022). The A-model and the P-model overestimates the increase in $A_{max}$ and A (Fig. 3B and C). However, the M-model predicts that LMA decreases with increasing $C_{a}$ which is different from the meta-analysis results (Fig. 3D).

**Figure 4.**
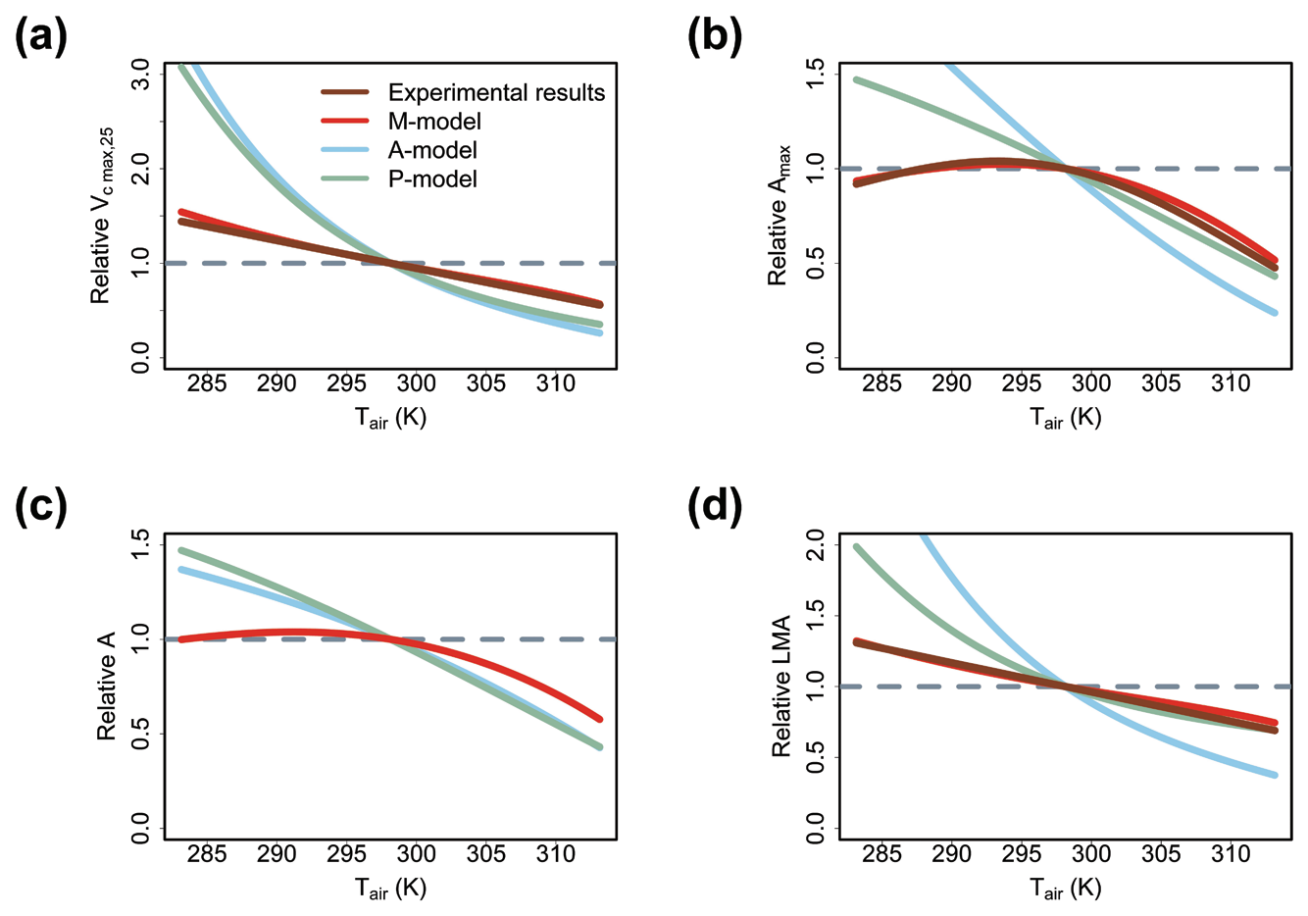
Predicted $V_{c\;\max,25}$, $A_{\max}$, A and LMA in relation to growth air temperature ($T_{air}$): Predicting the acclimation trends of (A) $V_{c\;max,25}$, (B) $A_{max}$, (C) A and (D) LMA to growth air temperature ($T_{air}$). We assumed that $T_{air}$ is equal to the leaf temperature. The experimental results come from Scafaro *et al.* (2017) and Cai *et al.* (2020). However, the data for A are not available in the two studies.

For growth air temperature ($T_{air}$), the three models all predicted that $V_{c\;max,25}$ and LMA increase with decreasing $T_{air}$ (Fig. 4A and D). The prediction of $V_{c\;max,25}$ and LMA by the M-model shows the highest consistency with the experimental data (Fig. 4A and D). The $V_{c\;max,25}$ and LMA of leaves grown in low$\;T_{air}$ are overestimated by the A-model and the P-model (Fig. 4A and D). The A-model and the P-model predicted that $A_{max}$ and A increase with decreasing $T_{air}$ (Fig. 4B and C). The M-model predicted that $A_{max}$ and A increase first and then decrease with decreasing $T_{air}$, which are consistent with the experimental data (Fig. 4B and C).

### Predicting the $V_{c\;max,25}$ of leaves grown in different light environments quantitatively

**Figure 5.**
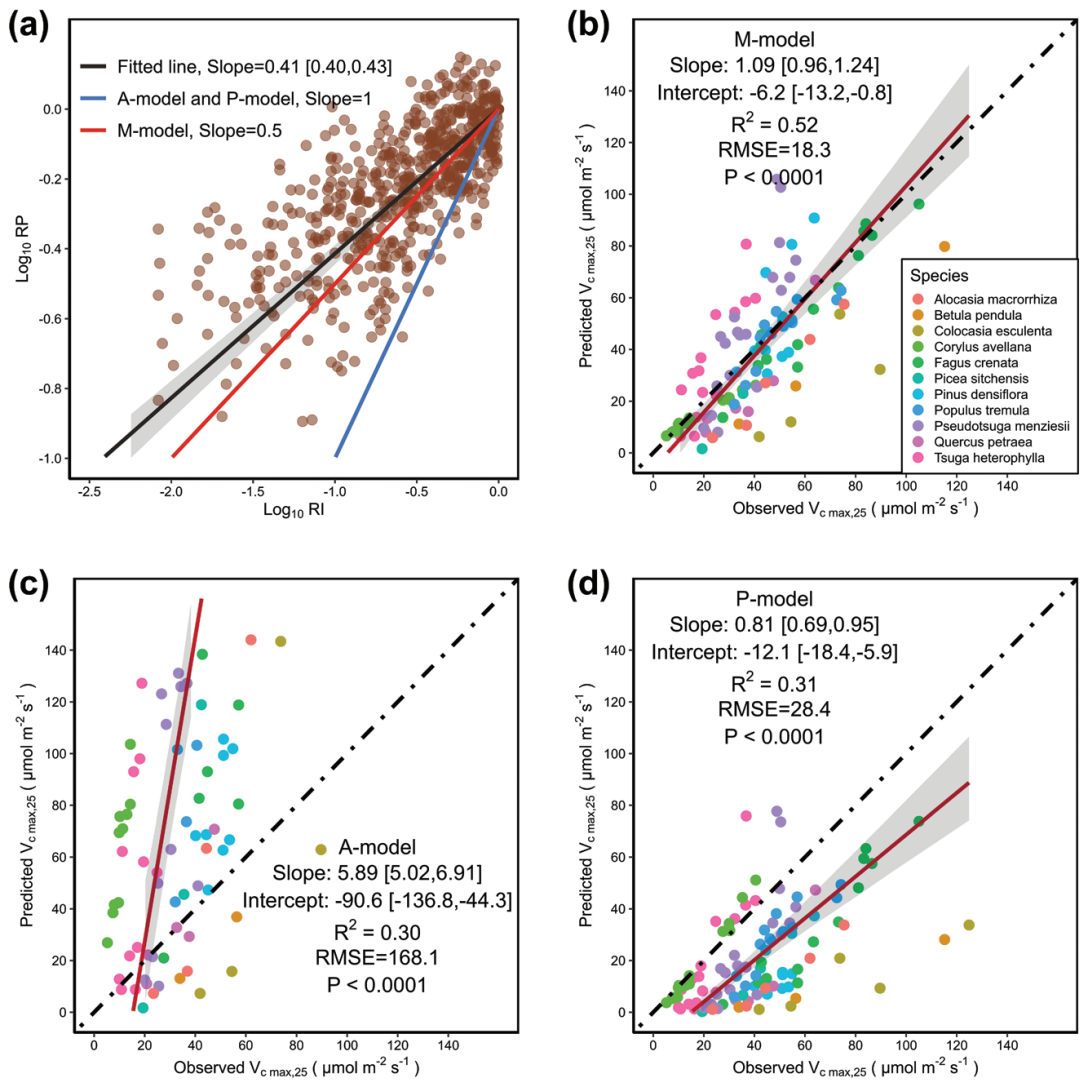
Predicting the relative and the actual $V_{c\;\max,25}$ of leaves grown in different light environments: (A) The relationship between the relative $V_{c\;max,25}$ (RP) and the relative light intensity (RI) (taking the logarithmic values of RP and RI). The numbers in brackets and grey region represent the 95% confidence interval. The black solid line was fitted by the standard major axis regression. (B), (C) and (D) were the comparison of the predicted $V_{c\;max,25}$ to the observed $V_{c\;max,25}$ of the M-model (B), the A-model (C) and the P-model (D), respectively. The red solid lines were the best-fit line from the standard major axis regression. The grey region represents the 95% confidence interval. The dotted black line is a 1:1 line.

For the prediction of the power–law relationship between the relative photosynthetic capacities per unit leaf area (RP) and the relative growth light intensity (RI), the A-model and the P-model both predict that RP is proportional to the 1 power of RI (Fig. 5A, blue solid line). The M-model predicts that RP is proportional to the 0.5 power of RI (Fig. 5A, red solid line). The actual data fitting result showed that RP is proportional to the 0.41 [0.40, 0.43] power of RI (Fig. 5A, black solid line), which is closer to the predicted results of the M-model.

For the prediction of the actual $V_{c\;max,25}$ of leaves grown in different light environments, the slope and the intercept of the relationships between the observed $V_{c\;\;\;max,25}$ and the $V_{c\;\;\;max,25}$ predicted by the M-model had 95% confidence intervals that bracketed 1 and 0, respectively (Fig. 5B). The slope and the intercept of the A-model or the P-model did not have 95% confidence intervals that bracketed 1 and 0, respectively (Fig. 5C and D). Among the three models, the prediction of the M-model exhibits the lowest root mean square error (RMSE), which indicates that its prediction is closest to the observed values. The M-model captures the most variation in $V_{c\;\;\;max,25}$ ($R^{2}$, 52%), which is significantly greater than the A-model (30%) and the P-model (31%).

We noticed that although the A-model and the P-model which both adopt the area-based optimality exhibit similar photosynthetic acclimation trends (Fig. 2 and Fig. 4), they have significant differences in predicting the actual value of $V_{c\;max,25}$ (Fig. 5C and D). This is because the P-model only assumes the existence of a cost directly proportional to photosynthetic capacity and ignores its physiological basis. The coefficient between the cost and the photosynthetic capacity was obtained by adjusting parameters to make the predicted values close to the observed values (Wang *et al.* 2017, 2023; Smith *et al.* 2019). The A model assumes that the costs mainly come from leaf respiration and leaf construction. The cost coefficients were obtained from previous measurements (Equation (12) and Equation (14)). Therefore, parameter adjustments may result in the differences between the A-model and the P-model in predicting $V_{c\;max,25}$.

### Predicting the leaf lifespan of leaves grown in different light environments

**Figure 6.**
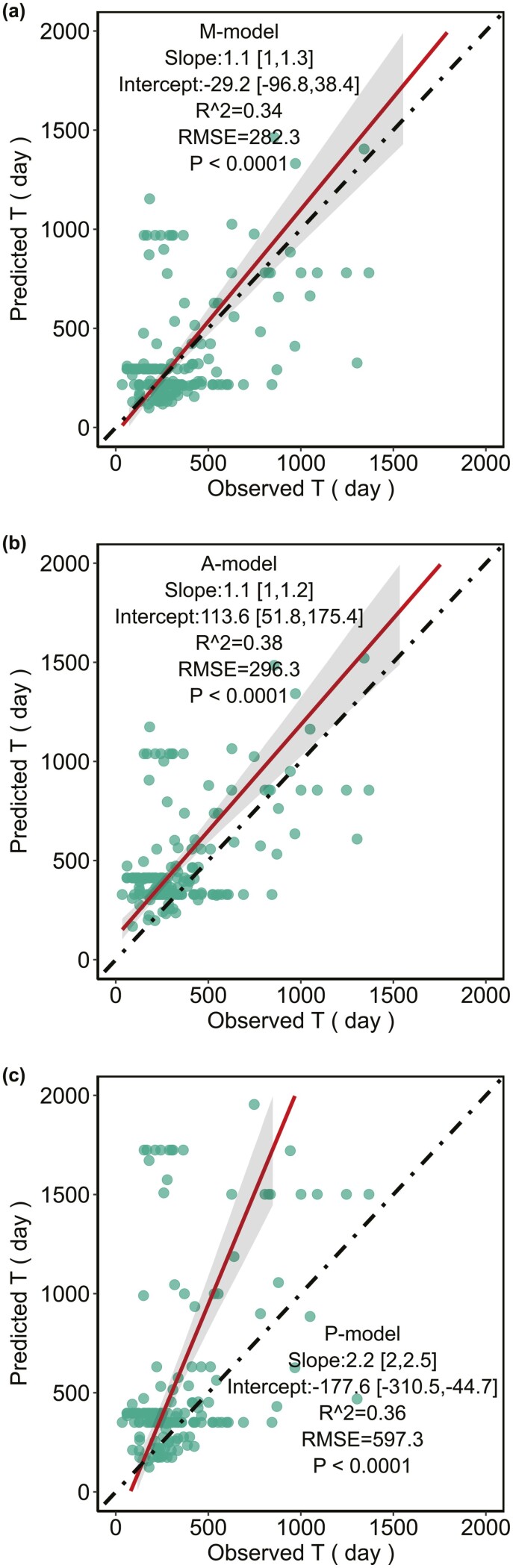
Predicting the leaf lifespan of leaves grown in different light environments: (A), (B) and (C) were the comparison of the predicted leaf lifespan (T) to the observed T of the M-model (A), the A-model (B) and the P-model (C), respectively. The red solid lines were the best-fit line from the standard major axis regression. The grey region represents the 95% confidence interval. The dotted black line is a 1:1 line.

The three models all predict that leaf lifespan (T) decreases as the growth light intensity increases, which is consistent with the observed data [see Supporting Information—Supplementary Fig. S1]. The slope and the intercept of the relationships between the observed T and the T predicted by the M-model had 95% confidence intervals that bracketed 1 and 0, respectively (Fig. 6A), while the T predicted by the A-model or the P-model did not (Fig. 6B and C). The prediction of the M-model has the lowest RMSE among the three models.

## Discussion

Our results suggest that the mass-based optimality may be a viable method to improve the predictive performance of optimization models. Optimization models have been used in vegetation and land surface models to improve the estimation of global carbon fluxes (Haxeltine and Prentice 1996; Franklin *et al.* 2020; Jiang *et al.* 2020; Luo and Keenan 2020; Harrison *et al.* 2021). Therefore, the improvement of photosynthetic optimization models by the mass-based optimality may also benefit these applications.

### Why do the M-model and the A-model predict differently?

**Figure 7.**
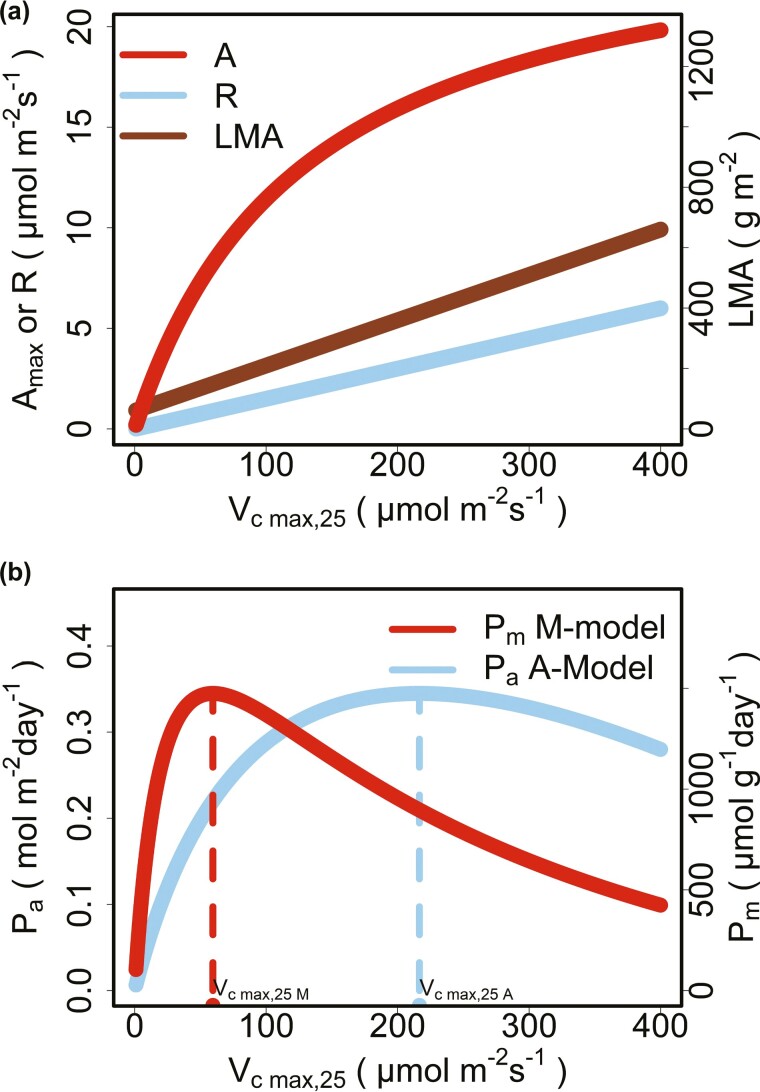
The changes in A, R, LMA, $P_{a}$ and $P_{m}$ as $V_{c\;max,25}$ increases: (A) The changes in A, R and LMA as $V_{c\;max,25}$ increases. (B) The changes in $P_{a}$ and $P_{m}$ as $V_{c\;max,25}$ increases.

The A-model and the M-model aim to adjust $V_{c\;max,25}$ to maximize the net photosynthetic carbon gain per unit leaf area per unit time ($P_{a}$) or per unit leaf dry mass per unit time ($P_{m}$), over the entire lifespan. Increasing $V_{c\;max,25}$ increases the photosynthetic rate per unit leaf area (A), and then facilitates the increase in $P_{a}$ and $P_{m}$. But limited by light intensity, the increase in A would gradually slow down (Fig. 7A). Increasing $V_{c\;max,25}$ would also have the negative effects on $P_{a}$ and $P_{m}$ because of the increase in leaf dry mass per unit leaf area (LMA), leaf construction cost per unit leaf area ($C_{c}$) and leaf respiration rate per unit leaf area (R). The reasons for the increase in $V_{c\;max,25}$ are generally due to thicker leaf mesophyll tissue (e.g. more layers or larger volumes of leaf mesophyll cells) and more photosynthetic substances (e.g. Rubisco) (Givnish 1988; Niinemets and Tenhunen 1997; Niinemets *et al.* 1999, 2006; Frak 2002; Niinemets 2007; Tong and Ng 2008; Buckley *et al.* 2013; Mathur *et al.* 2018). More leaf mesophyll tissue and photosynthetic substances would result in the increase in LMA (John *et al.* 2017) and $C_{c}$ (Barthod and Epron 2005) (Fig. 7A). More leaf mesophyll cell and protein turnover increase R (Wang *et al.* 2020) (Fig. 7A). Leaves need to balance the benefits (A) and costs (R, LMA and $C_{c}$) of $V_{c\;max,25}$ to maximizes $P_{a}$ or $P_{m}$ (Fig. 7B). More specially, with $V_{c\;max,25}$ increasing, the increase in A gradually slows down while R and $C_{c}$ increases linearly (Fig. 7A), resulting in $P_{a}$ first increasing and then beginning to decrease at $V_{c\;max,25\;A}$ (Fig. 7B). Due to LMA increases linearly with $V_{c\;max,25}$ (Fig. 7A), $P_{m}$, which is $P_{a}$ divided by LMA (Equation (2), $P_{m}=P_{a}/LMA$), would begin to decrease at a lower $V_{c\;max,25}$ (i.e. $V_{c\;max,25\;M}$) than $V_{c\;max,25\;A}$ (Fig. 7B). As a result, the A-model and the M-model make different predictions for the optimal $V_{c\;max,25}$ (i.e. $V_{c\;max,25\;A}$ and $V_{c\;max,25\;M}$) and $V_{c\;max,25\;M}$ is generally lower than $V_{c\;max,25\;A}$.

### Why does the M-model perform better than the A-model?

The relationship between plant growth and the net photosynthetic carbon assimilation theoretically explains why photosynthetic acclimation maximizes $P_{m}$ rather than $P_{a}$. Plants need to effectively utilize their limited resources to construct leaves and obtain more photosynthetic products (Givnish 1988; Lambers *et al.* 2008). The net photosynthetic carbon assimilation per unit leaf construction cost, which reflects the efficiency of leaves in utilizing dry matter, is an important determinant of plant growth rate (Givnish 1988; Poorter *et al.* 1990; Garnier 1991; Lambers *et al.* 2008). Leaf construction cost per unit leaf dry mass is relatively conservative, while leaf construction cost per unit leaf area varies greatly among different growth environments (Bazzaz and Grace 1997; Barthod and Epron 2005; Poorter *et al.* 2006; Villar *et al.* 2006; Feng *et al.* 2008). Therefore, $P_{m}$ better represents the net photosynthetic carbon assimilation per unit leaf construction cost and then is more closely related to plant growth (Givnish 1988). The theoretical modelling of plant relative growth rate (RGR) intuitively displays this point (Lambers and Poorter 1992; Bazzaz and Grace 1997; Lambers *et al.* 2008):


$$\begin{array}{l} RGR=\frac{\left(\frac{A}{LMA}-LR\right)\times LMR-SR\times SMR-RR\times RMR}{C_{c}}, \end{array}$$

where A is the photosynthetic rate per unit leaf area, LMA is leaf dry mass per unit leaf area, LR, SR and RR are the respiration rate per unit leaf, stem and root dry mass respectively, LMR, SMR and RMR are the fractions of biomass allocated to leaves, stems and roots, respectively, and $C_{c}$ is the construction costs per unit plant dry mass. Maximizing $P_{m}$ is equal to maximize (A/LMA)−LR, which contributes to increase RGR. Previous observations also support this point, finding that RGR is weakly correlated with the net assimilation rate per unit leaf area (Poorter *et al.* 1990; Garnier 1991) and strongly correlated with the net assimilation rate per unit leaf dry mass (Poorter *et al.* 1990; Garnier 1991; Lambers and Poorter 1992; Bazzaz and Grace 1997; Lambers *et al.* 2008). Thus, maximizing $P_{m}$ through photosynthetic acclimation may be more beneficial for growth than maximizing $P_{a}$. A higher relative growth rate helps individuals/leaves compete for light resources and produce more leaves to obtain more photosynthetic products. This, in turn, helps plants increase fitness and survive in natural selection (Lambers *et al.* 2008). Thus, plants that maximize $P_{m}$ through photosynthetic acclimation may have a competitive advantage over those plants that do not. Natural selection may favour plants that maximize $P_{m}$ rather than $P_{a}$ through photosynthetic acclimation. Therefore, the mass-based optimality may be a more reasonable assumption than the area-based optimality and improve the prediction effects of the M-model.

### The problems that warrant improvement of the M-model

(1) The M-model may overestimate $V_{c\;max,25}$ and $A_{max}$ in high light (Fig. 2A and B). The M-model predicted that $V_{c\;max,25}$ and $A_{max}$ would increase infinitely with increasing light intensity, instead of approaching a constant value (Fig. 2A and B). This was obviously unrealistic. This may be caused by the assumption that $T_{leaf}$ is equal to $T_{air}$. In fact, according to the leaf energy model (Okajima *et al.* 2012), high light would increase energy input to the leaves and then would increase $T_{leaf}$. This may slow down the increase in $V_{c\;max,25}$ and $A_{max}$ (Fig. 4A and B). High $T_{leaf}$ would lead to high vapour pressure deficit (VPD) and then cause stomatal closure (Medlyn *et al.* 2011), which further hinders the increase in $A_{max}$. In addition, high light stress may also have a negative effect on the increase in $A_{max}$ because light stress may lead to photoinhibition (Niinemets 2012). Therefore, this problem may be solved by considering the leaf energy balance model and light stress.
(2) The M-model predicted that LMA decreases with increasing growth CO_2_ concentration, which is different from the meta-analysis results (Fig. 3D). This is because the M-model only ignores the contribution of non-structural carbohydrates. In high CO_2_ concentration, the increase in LMA may be caused by the excessive accumulation of non-structural carbohydrates (e.g. starch) in leaves (Poorter *et al.* 2022). This problem may be solved by incorporating non-structural carbohydrates into the modelling of LMA.
(3) Accurate acquisition of the model parameters k and b for different species is difficult. Problems such as small data size and large measurement errors may affect the accurate estimation of k and b. This may hinder the application of the M-model. k and b may be related to leaf anatomical structure (Equation (13)). If the quantitative relationships between these parameters and leaf anatomical structures can be established, it may make it easier to obtain the values of k and b in future. An alternative solution is to ignore interspecific variation and only consider photosynthetic acclimation to environments. In this case, the median or the mean of existing k and b could be chosen as representative values to drive the model for rough prediction. However, this method ignores the interspecific variation in $V_{c\;max,25}$ and may reduce the model’s explanation to the variation in $V_{c\;max,25}$.
(4) The physiological mechanisms associated with leaf lifespan and senescence are unknown and existing uncertainty in related model parameter settings. For example, the relationship between $LL_{p}$ and $A_{n\;day}(0)/LMA$ (Equation (5)) was an empirical formula without a clear physiological mechanism. Besides, limited by data, the data used to fit this empirical formula mostly come from woody plants. It is not clear whether this formula works in herbaceous plants. Further research on leaf senescence and lifespan may help solve this problem. To simplify the models, referring to K. Kikuzawa’s original work (Kikuzawa 1991), ignoring the impact of non-growing seasons on the estimation of leaf lifespan. There has been a lot of discussion and studies on this problem in previous work (Kikuzawa 1991, 1995; Kikuzawa *et al.* 2013; Seki *et al.* 2015; Castorena *et al.* 2022). In future, the impacts of non-growing seasons can be considered to further improve the prediction of leaf lifespan.

## Supporting Information

The following additional information is available in the online version of this article –

Supplementary Figure 1. The acclimation of leaf lifespan to growth light intensity. (A) The observed leaf lifespan (T) of leaves grown in different growth light intensity (I). (B) Predicting the acclimation trends of leaf lifespan (T) to growth light intensity (I).

Supplementary Material 1. Verification dataset and analysis codes.

Supplementary Material 2. The relationship between LMA and $V_{c\;max,25}$.

Supplementary Material 3. The dataset about photosynthetic acclimation to light intensity.

Supplementary Material 4. Leaf senescence data.

Supplementary Material 5. Model derivation.

plae044_suppl_Supplementary_Figure

plae044_suppl_Supplementary_Material_S1

plae044_suppl_Supplementary_Material_S2

plae044_suppl_Supplementary_Material_S3

plae044_suppl_Supplementary_Material_S4

plae044_suppl_Supplementary_Material_S5

## Data Availability

The data that supports the findings of this study are available in the Supplementary Material of this article
