# Supplementary material for: The leaf-scale mass-based photosynthetic optimization model better predicts photosynthetic acclimation than the area-based
Source: AoB Plants. 2024 Aug 19;16(5):plae044. doi: 10.1093/aobpla/plae044 (PMC11459265; doi:10.1093/aobpla/plae044)

Supplementary Figure

Supplementary Figure 1. The acclimation of leaf lifespan to growth light intensity: (a) The observed leaf lifespan (T) of leaves grown in different growth light intensity (I). (b) Predicting the acclimation trends of leaf lifespan (T) to growth light intensity (I).

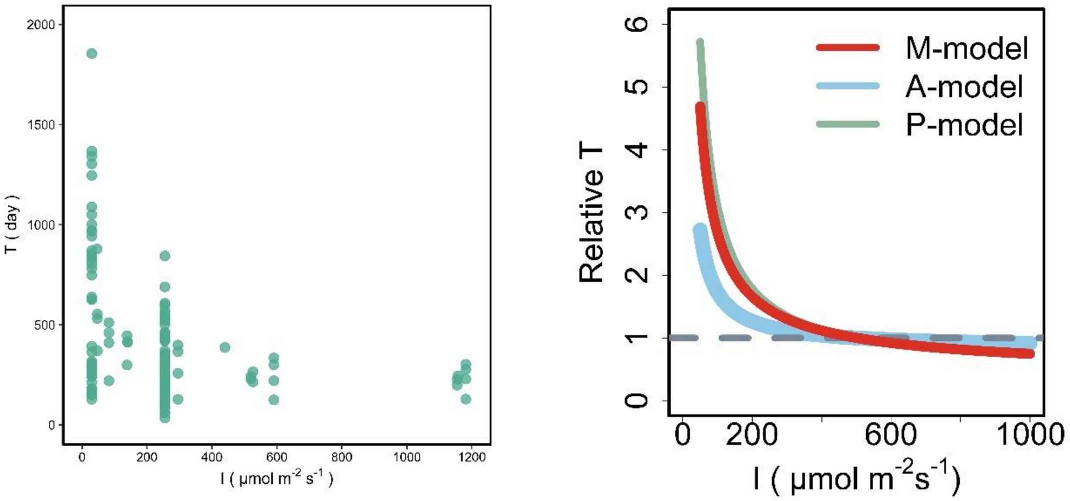

Supplement: plae044_suppl_Supplementary_Figure [file plae044_suppl_supplementary_figure.pdf]
