# Supplementary figures and images for: The leaf-scale mass-based photosynthetic optimization model better predicts photosynthetic acclimation than the area-based
Source: AoB Plants. 2024 Aug 19;16(5):plae044. doi: 10.1093/aobpla/plae044 (PMC11459265; doi:10.1093/aobpla/plae044)

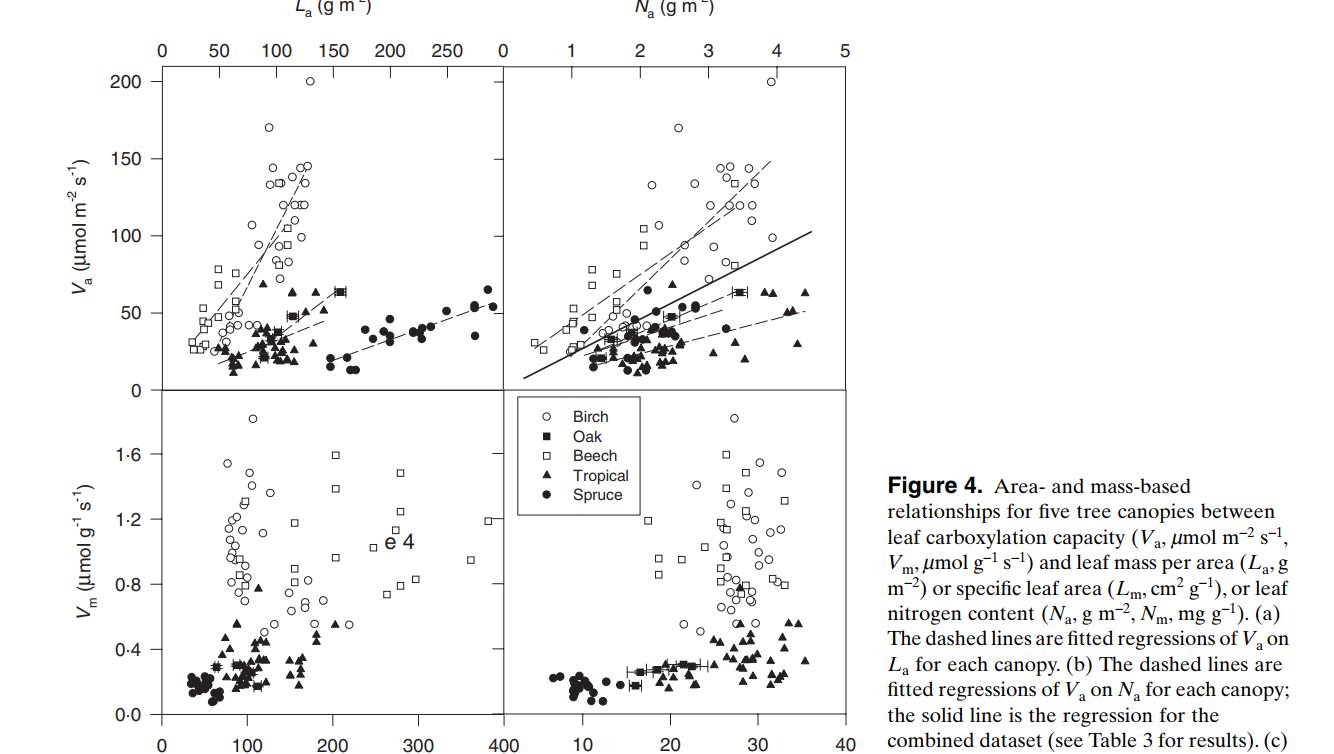

Supplement: plae044_suppl_Supplementary_Material_S2 [file plae044_suppl_supplementary_material_s2.zip › Supplementary Material 2 LMA=kVcmax+b/Raw picture LMA=kVcmax25+b/Acclimation of photosynthetic capacity to irradiance in tree.JPG]

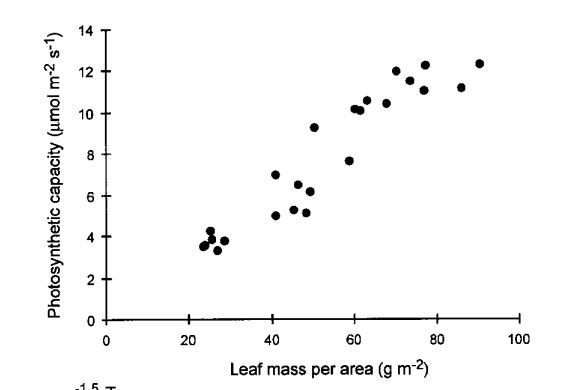

Supplement: plae044_suppl_Supplementary_Material_S2 [file plae044_suppl_supplementary_material_s2.zip › Supplementary Material 2 LMA=kVcmax+b/Raw picture LMA=kVcmax25+b/Canopy photosynthesis of sugar maple (Acer saccharum)comparing.JPG]

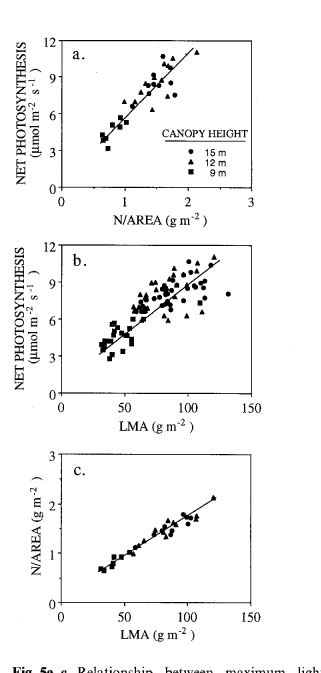

Supplement: plae044_suppl_Supplementary_Material_S2 [file plae044_suppl_supplementary_material_s2.zip › Supplementary Material 2 LMA=kVcmax+b/Raw picture LMA=kVcmax25+b/Canopy structure and vertical patterns of photosynthesis.JPG]

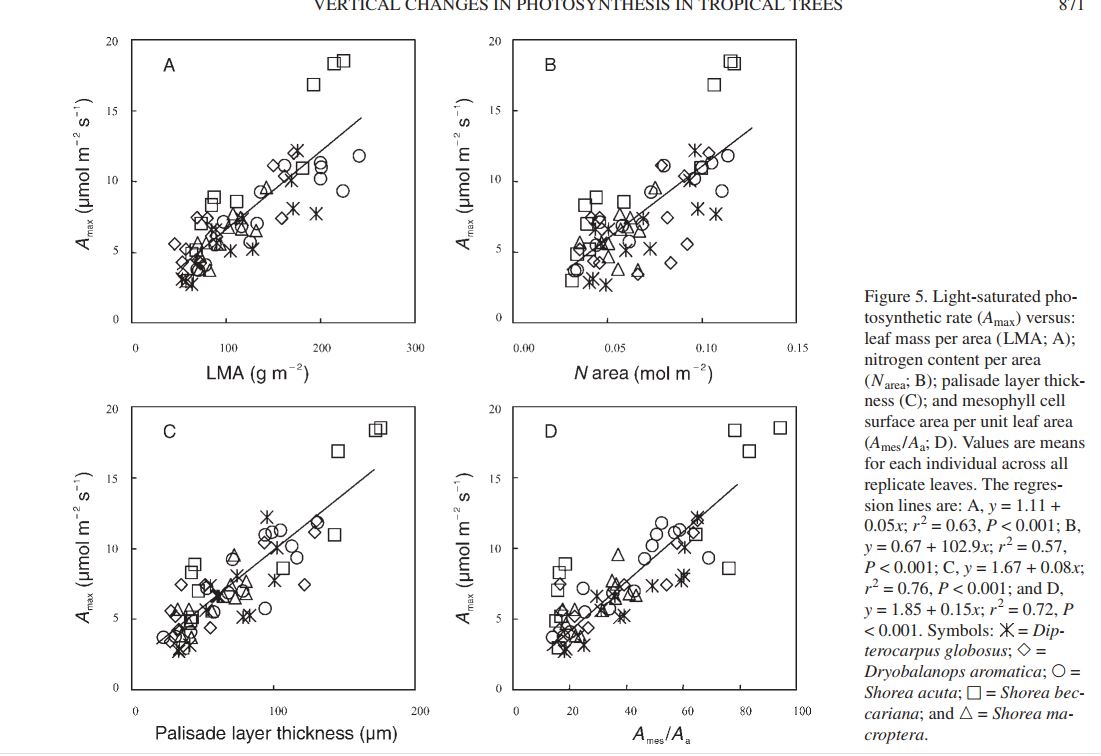

Supplement: plae044_suppl_Supplementary_Material_S2 [file plae044_suppl_supplementary_material_s2.zip › Supplementary Material 2 LMA=kVcmax+b/Raw picture LMA=kVcmax25+b/Changes in photosynthesis and leaf characteristics with tree height in.JPG]

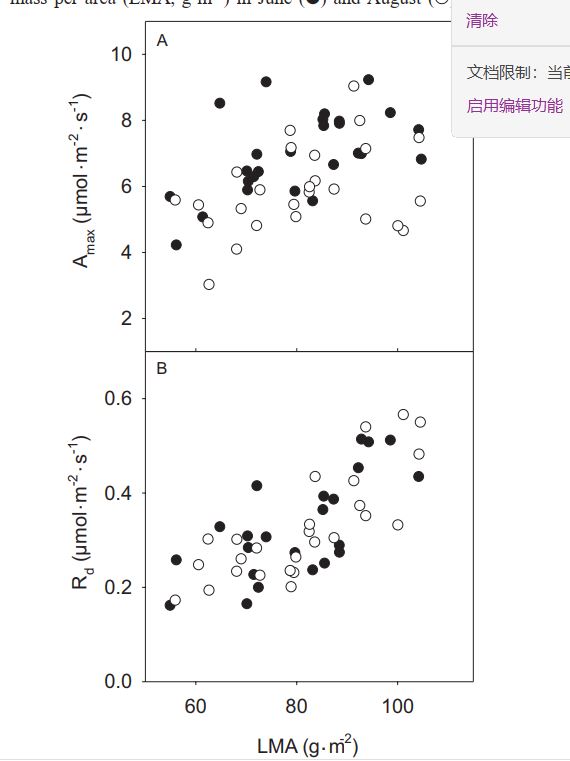

Supplement: plae044_suppl_Supplementary_Material_S2 [file plae044_suppl_supplementary_material_s2.zip › Supplementary Material 2 LMA=kVcmax+b/Raw picture LMA=kVcmax25+b/Evaluation of the importance of acclimation of.JPG]

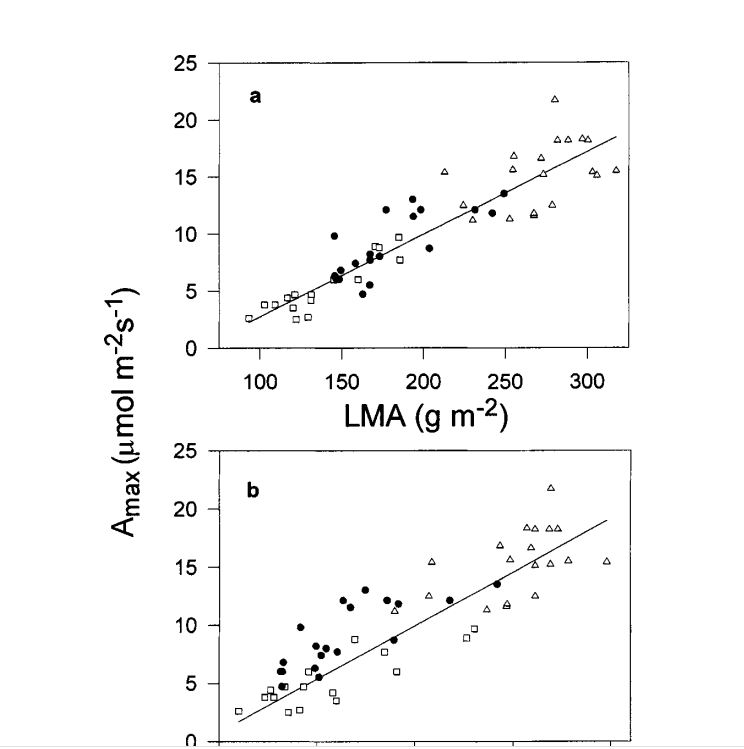

Supplement: plae044_suppl_Supplementary_Material_S2 [file plae044_suppl_supplementary_material_s2.zip › Supplementary Material 2 LMA=kVcmax+b/Raw picture LMA=kVcmax25+b/Foliage physiology and biochemistry in response.JPG]

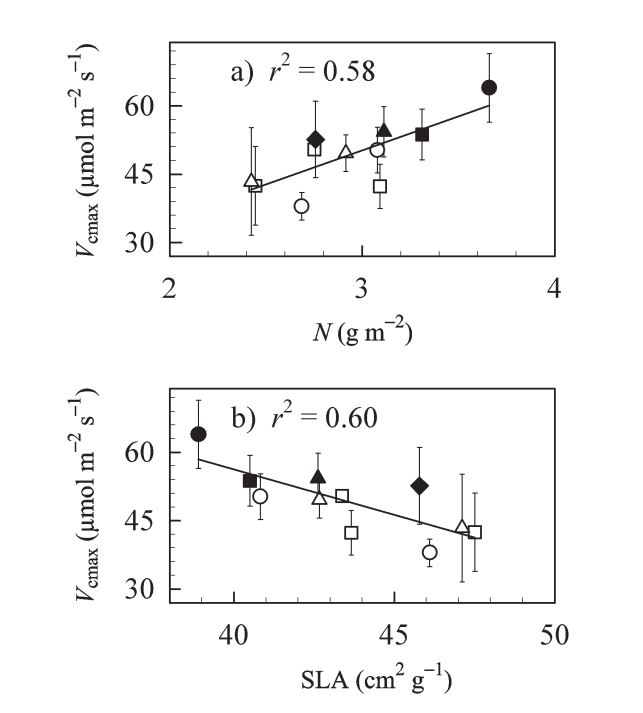

Supplement: plae044_suppl_Supplementary_Material_S2 [file plae044_suppl_supplementary_material_s2.zip › Supplementary Material 2 LMA=kVcmax+b/Raw picture LMA=kVcmax25+b/Horizontal and vertical variations in photosynthetic capacity in a Pinus.JPG]

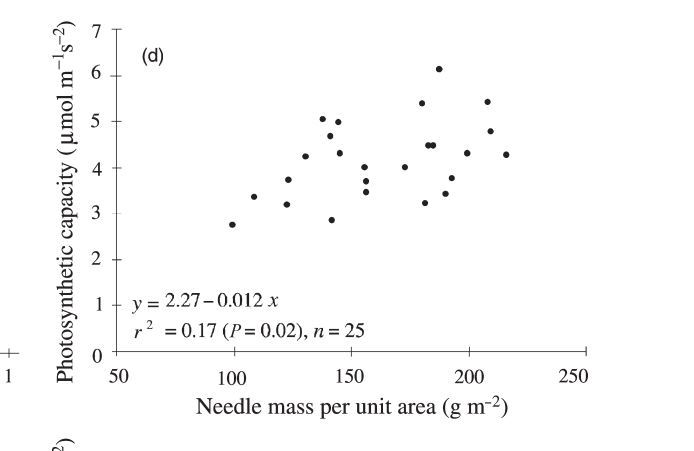

Supplement: plae044_suppl_Supplementary_Material_S2 [file plae044_suppl_supplementary_material_s2.zip › Supplementary Material 2 LMA=kVcmax+b/Raw picture LMA=kVcmax25+b/Importance of needle age and shoot structure on canopy net.JPG]

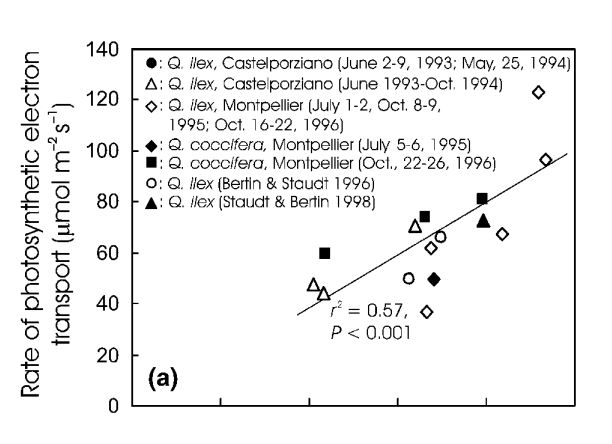

Supplement: plae044_suppl_Supplementary_Material_S2 [file plae044_suppl_supplementary_material_s2.zip › Supplementary Material 2 LMA=kVcmax+b/Raw picture LMA=kVcmax25+b/Monoterpene emissions in relation to foliar photosynthetic .JPG]

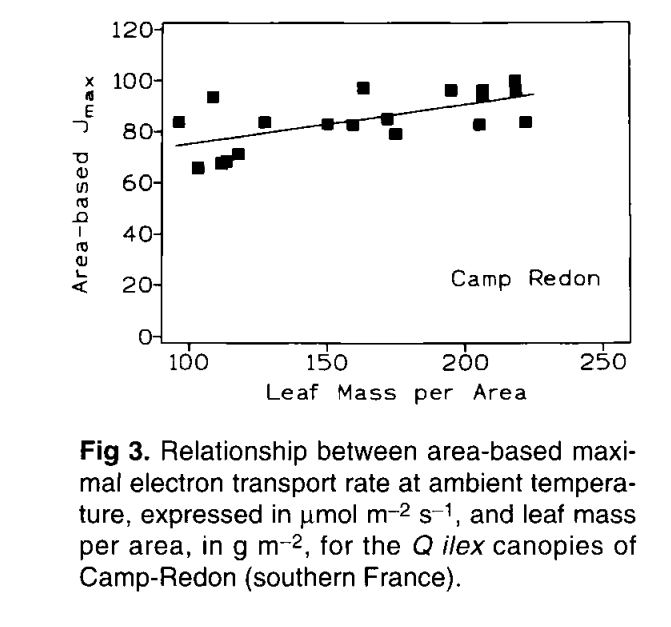

Supplement: plae044_suppl_Supplementary_Material_S2 [file plae044_suppl_supplementary_material_s2.zip › Supplementary Material 2 LMA=kVcmax+b/Raw picture LMA=kVcmax25+b/Optimization of carbon gain in canopies of Mediterranean.JPG]

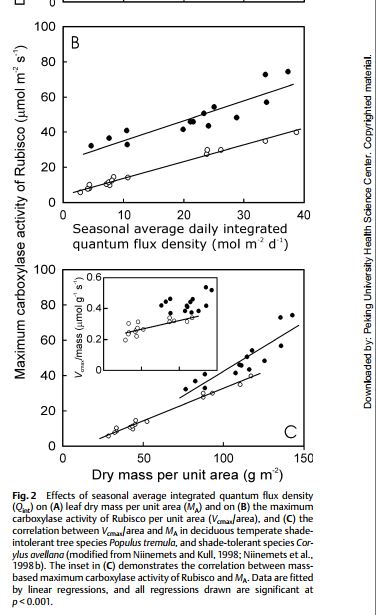

Supplement: plae044_suppl_Supplementary_Material_S2 [file plae044_suppl_supplementary_material_s2.zip › Supplementary Material 2 LMA=kVcmax+b/Raw picture LMA=kVcmax25+b/Photosynthetic Acclimation to Simultaneous and Interacting.JPG]

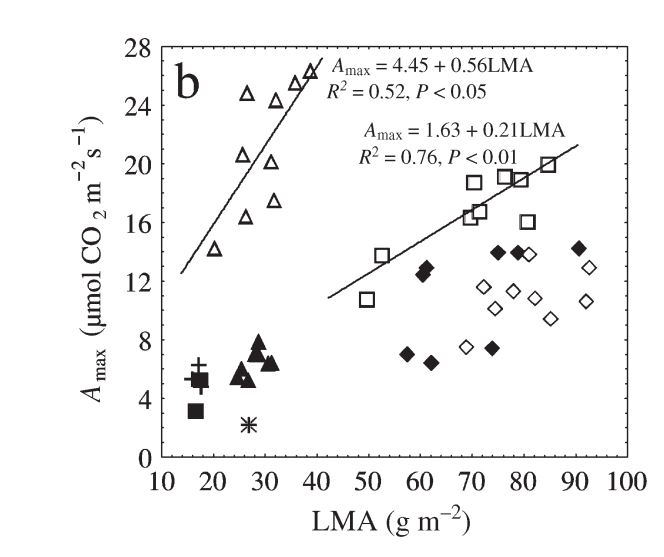

Supplement: plae044_suppl_Supplementary_Material_S2 [file plae044_suppl_supplementary_material_s2.zip › Supplementary Material 2 LMA=kVcmax+b/Raw picture LMA=kVcmax25+b/Photosynthetic capacity in relation to nitrogen in the canopy of a Quercus robur,.JPG]

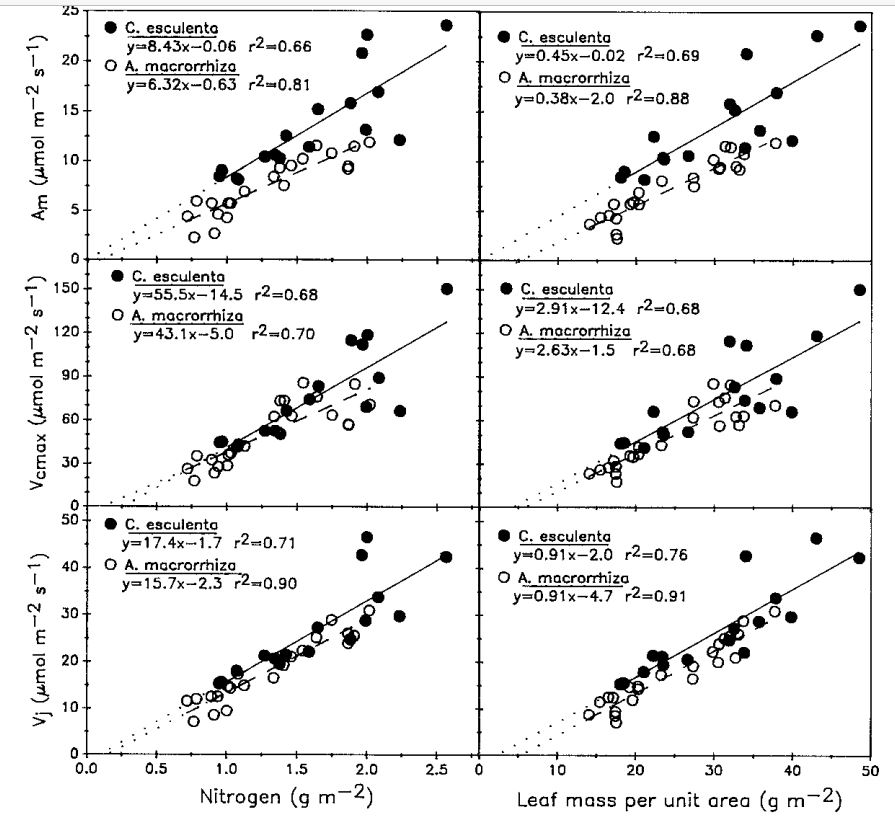

Supplement: plae044_suppl_Supplementary_Material_S2 [file plae044_suppl_supplementary_material_s2.zip › Supplementary Material 2 LMA=kVcmax+b/Raw picture LMA=kVcmax25+b/Photosynthetic characteristics of a tropical forest understory herb, Alocasia macrorrhiza, and a related crop species, Colocasia esculenta grown in contrasting light environments.JPG]

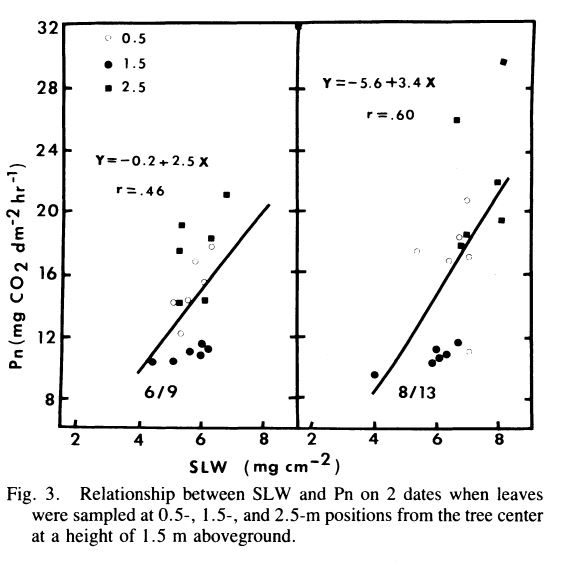

Supplement: plae044_suppl_Supplementary_Material_S2 [file plae044_suppl_supplementary_material_s2.zip › Supplementary Material 2 LMA=kVcmax+b/Raw picture LMA=kVcmax25+b/Seasonal Changes in Specific Leaf Weight,.JPG]

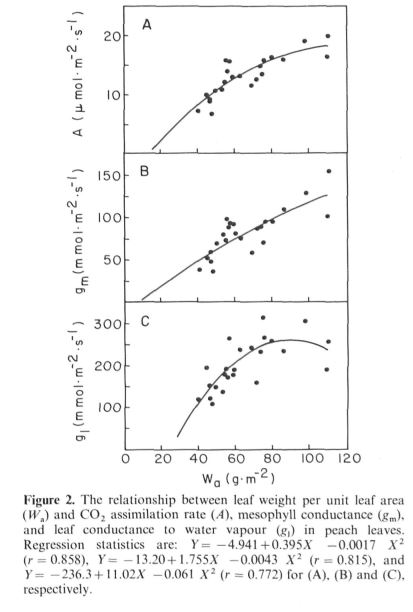

Supplement: plae044_suppl_Supplementary_Material_S2 [file plae044_suppl_supplementary_material_s2.zip › Supplementary Material 2 LMA=kVcmax+b/Raw picture LMA=kVcmax25+b/Seasonal relationships between leaf nitrogen.JPG]

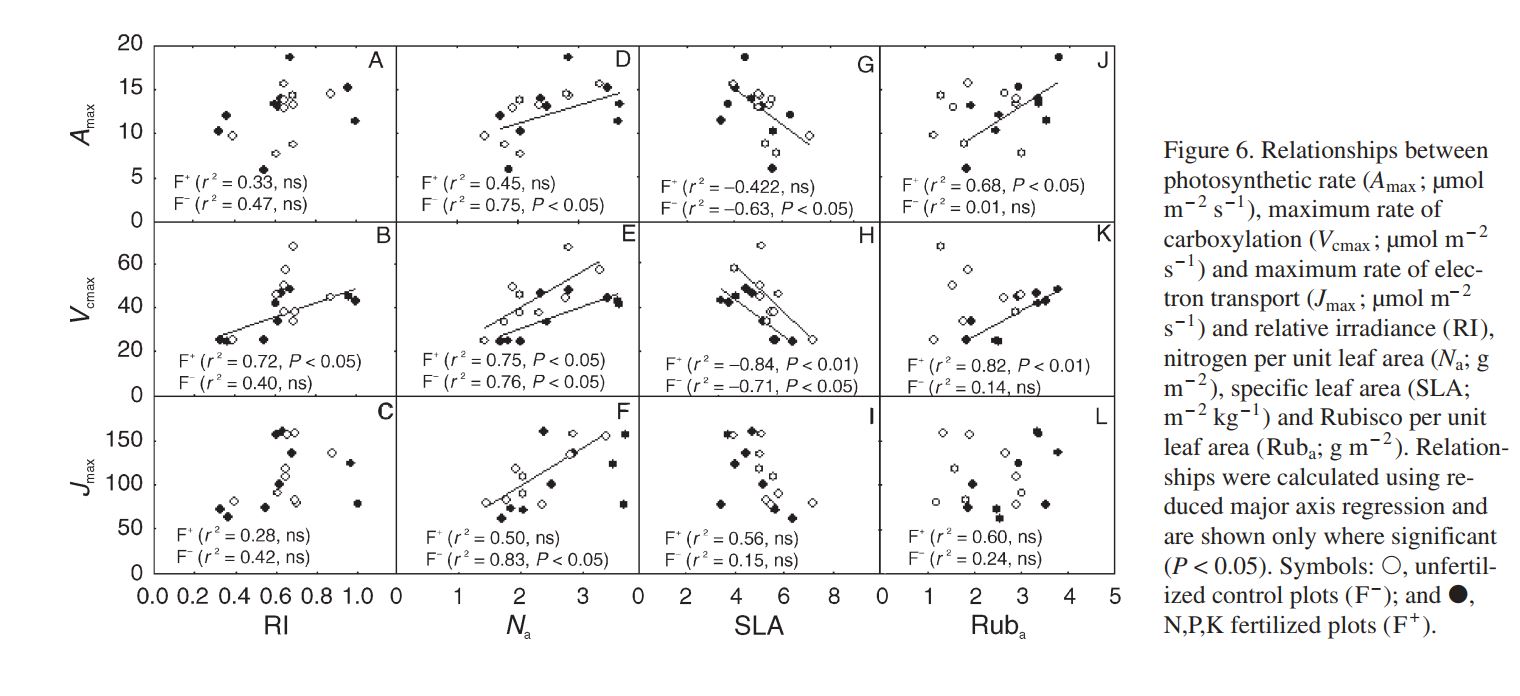

Supplement: plae044_suppl_Supplementary_Material_S2 [file plae044_suppl_supplementary_material_s2.zip › Supplementary Material 2 LMA=kVcmax+b/Raw picture LMA=kVcmax25+b/Within-canopy nitrogen and photosynthetic gradients are unaffected.JPG]
