# Supplementary material for: The leaf-scale mass-based photosynthetic optimization model better predicts photosynthetic acclimation than the area-based
Source: AoB Plants. 2024 Aug 19;16(5):plae044. doi: 10.1093/aobpla/plae044 (PMC11459265; doi:10.1093/aobpla/plae044)
Supplement: plae044_suppl_Supplementary_Material_S5 [file plae044_suppl_supplementary_material_s5.pdf]

Supplementary Material 5

The specific modelling process of A

According to the right-angle hyperbola formula of light response curve, A ($\mu mol\ C\ m^{-2}\ s^{-1}$) is:

$$A = \frac{\alpha I A_{max}}{\alpha I + A_{max}}, \qquad (S1)$$

where A_{max} ($\mu mol\ C\ m^{-2}\ s^{-1}$) is the light-saturated photosynthetic rate, α is the apparent quantum yield and I is photosynthetic photon flux density ($\mu mol\ m^{-2}\ s^{-1}$).

(1) Modelling A_{max}

When light is saturated, the carboxylation rate of Rubisco and the potential electron transfer rate reach their maximum values, i.e., the maximum carboxylation rate of Rubisco ($V_{c\,max}$, $\mu mol\ CO_2\ m^{-2}\ s^{-1}$) and the maximum potential electron transport rate (J_{max}, $\mu mol\ m^{-2}\ s^{-1}$). According to FvCB model, A_{max} may be limited by the activity of Rubisco and RuBP regeneration (Farquhar et al. 1980):

$$A_{\max C} = V_{c\,max} \cdot \frac{C_{ch} - \Gamma^*}{C_{ch} + K}, \qquad (S2)$$

$$A_{\max J} = \frac{J_{max}}{4} \cdot \frac{C_{ch} - \Gamma^*}{C_{ch} + 2\Gamma^*}, \qquad (S3)$$

$$A_{max} = \min(A_{\max C}, A_{\max J}), \qquad (S4)$$

where C_{ch} (ppm) is the chloroplastic CO_2 concentration, Γ^* (ppm) is the photosynthetic CO_2 compensation point without dark respiration and K (ppm) is the Michaelis-Menten constants. $V_{c\,max}$, J_{max}, Γ^* and K are influenced by leaf temperature:

$$V_{c\,max} = V_{c\,max,25} f_{V_{c\,max}}(T_{leaf}), \qquad (S5)$$

$$J_{max} = J_{max,25} f_{J_{max}}(T_{leaf}), \qquad (S6)$$

where $V_{c\,max,25}$ ($\mu mol\ CO_2\ m^{-2}\ s^{-1}$) is the maximum carboxylation rate of Rubisco at 25 °C, $J_{max,25}$ ($\mu mol\ m^{-2}\ s^{-1}$) is the maximum potential electron transport rate at 25 °C and $f_{V_{c\,max}}$ and $f_{J_{max}}$ are the leaf temperature dependent function of $V_{c\,max}$ and J_{max} respectively. T_{leaf} was assumed to be equal to growth air temperature (T_{air}, °C).

The detailed information about the leaf temperature dependence of $f_{V_{c\,max}}$, $f_{J_{max}}$, Γ^* and K can be found in the next section (Bernacchi et al. 2003, Kattge and Knorr 2007).

In nature, $V_{c\,max,25}$ and $J_{max,25}$ are coordinated. Previous studies showed that:

$$J_{max,25} = r_{J,V} V_{c\,max,25}, \tag{S7}$$

$$r_{J,V} = 2.59 - 0.035 T_{air}, \tag{S8}$$

where $r_{J,V}$ is the ratio of $J_{max,25}$ to $V_{c\,max,25}$ and T_{air} is the growth air temperature (T_{air}, °C).

Substituting Eq. (S6) and Eq. (S7) into Eq. (S3), and Substituting Eq. (S5) into Eq. (S2), A_{max} is:

$$A_{max} = V_{c\,max,25} \cdot \min\left(\frac{f_{V_{c\,max}}(C_{ch} - \Gamma^*)}{C_{ch} + K}, \frac{r_{J,V} f_{J_{max}}(C_{ch} - \Gamma^*)}{4C_{ch} + 8\Gamma^*}\right). \tag{S9}$$

To simplify the formula writing, we set:

$$f_V = \min\left(\frac{f_{V_{c\,max}}(C_{ch} - \Gamma^*)}{C_{ch} + K}, \frac{r_{J,V} f_{J_{max}}(C_{ch} - \Gamma^*)}{4C_{ch} + 8\Gamma^*}\right). \tag{S10}$$

(2) Modelling $\boldsymbol{\alpha}$

According to FvCB model, the photosynthetic rate in low light (A_j, $\mu mol\ C\ m^{-2}\ s^{-1}$) is limited by RuBP regeneration:

$$A_j = \frac{J}{4} \cdot \frac{C_{ch} - \Gamma^*}{C_{ch} + 2\Gamma^*}, \tag{S11}$$

$$J = \frac{\alpha_j \cdot I + J_{max} - \sqrt{\left(\alpha_j \cdot I + J_{max}\right)^2 - 4\theta_j \cdot \alpha_j \cdot I \cdot J_{max}}}{2\theta_j}, \tag{S12}$$

$$\alpha_j = abs \cdot R_{PSII} \cdot \Phi_{PSII} \cdot (1 - l), \tag{S13}$$

where J ($\mu mol\ m^{-2}\ s^{-1}$) is the electron transport rate and J_{max} ($\mu mol\ m^{-2}\ s^{-1}$) is the maximum electron transport rate. abs is the absorbance of photosynthetic photon flux density by leaves, which can be set as 0.84. R_{PSII} is the proportion of captured light energy allocated to photosystem II in low light, which can be set as 0.5 in low light. l is the spectral correction factor of white light (set as 0.15). Φ_{PSII} is the maximum quantum yield of photosystem II, which can be set as 0.8. θ_j is the convexity factors of non-rectangular hyperbolas (Laĭsk et al. 2009).

In low light approach darkness, J is basically not limited by J_{max} and is proportional to light intensity:

$$J \approx abs \cdot R_{PSII} \cdot \Phi_{PSII} \cdot (1-l) \cdot I. \qquad (S14)$$

The initial slope of light-response curve in low light (i.e., α) is:

$$\alpha = \frac{A_j}{I} = \frac{abs \cdot R_{PSII} \cdot \Phi_{PSII} \cdot (1-l)}{4} \cdot \frac{C_{ch} - \Gamma^*}{C_{ch} + 2\Gamma^*}. \qquad (S15)$$

(3) Modelling C_{ch}

C_{ch} is related to stomatal conductance and mesophyll conductance. Many models have been proposed to predict stomatal conductance to CO_2 (g_{sc}, $mol/m^2/s$). Some based on experience (Ball et al. 1987, Leuning 1995) and some based on the principle of optimality (Medlyn et al. 2011, Prentice et al. 2014). Although their theoretical bases are different, their final formula forms were similar:

$$g_{sc} = g_0 + \left(1 + \frac{g_1}{\sqrt{VPD}}\right)\frac{A}{C_a}, \qquad (S16)$$

where g_0 ($mol/m^2/s$) is the residual conductance and g_1 (${kP_a}^{0.5}$) is the model fitted parameter which is different among different species. A ($\mu mol/m^2/s$) is the photosynthetic rate per unit leaf area. VPD (kP_a) is the leaf vapor pressure deficit. Here, g_0 is set as 0 $mol/m^2/s$ and g_1 is set as 3.6 ${kP_a}^{0.5}$, which are the average value of different species. (Medlyn et al. 2011).

Mesophyll conductance to CO_2 (g_m, $mol/m^2/s$) varies among species and can also response to environmental factors just like g_{sc}. Modelling mesophyll conductance is difficult because a lack of understanding of its physiological mechanisms. Currently, there is no good mesophyll conductance model available and g_m generally can only be introduced into models through special assumptions (Buckley et al. 2013). Some studies have observed that g_{sc} and g_m responded in parallel to irradiance, CO_2, temperature, and drought stress (Xiong et al. 2018). Therefore, g_m is assumed to be proportional to g_{sc} (Xiong et al. 2018):

$$g_m = 1.12 g_{sc}, \qquad (S17)$$

where 1.12 is a coefficient obtained by fitting the data collected by Li Yong et al (Li Yong et al. 2013). A, g_{sc} and g_m have the following relationships:

$$A \approx g_{sc}(C_a - C_i) = g_m(C_i - C_{ch}), \qquad (S18)$$

where C_a (ppm) is the atmospheric CO_2 (ppm) concentration, C_i (ppm) is the intercellular CO_2 concentration and C_{ch} is the chloroplastic CO_2 concentration. So,

C_{ch} is:

$$C_{ch} = \frac{g_1 - 0.89\sqrt{VPD}}{g_1 + \sqrt{VPD}} C_a. \qquad (S19)$$

(4) The modelling results of *A*

Substituting Eq. (S9) and Eq. (S19) into Eq. (S1), A is:

$$A = \frac{\alpha I V_{c\,max,25} f_V}{\alpha I + V_{c\,max,25} f_V}. \qquad (S20)$$

The leaf temperature dependent functions of $f_{V_{c\,max}}$, $f_{J_{max}}$, Γ^*, K and f_R

The leaf temperature dependent functions of K, Γ^*, $f_{V_{c\,max}}$ and f_R come from previous studies(Bernacchi et al. 2003, Kattge and Knorr 2007, Heskel et al. 2016):

$$K = K_c\left(1 + \frac{O}{K_o}\right), \qquad (S21)$$

$$K_c = e^{38.05 - \frac{9553.42}{T_{leaf}}}, \qquad (S22)$$

$$K_o = e^{20.30 - \frac{4375.59}{T_{leaf}}}, \qquad (S23)$$

$$\Gamma^* = e^{19.02 - \frac{4549.99}{T_{leaf}}}, \qquad (S24)$$

$$f_{V_{c\,max}} = e^{\frac{H_{a\,V}(T_{leaf} - 298.15)}{298.15 T_{leaf} R}} \frac{1 + e^{\frac{298.15\Delta S_V - H_d}{298.15R}}}{1 + e^{\frac{T_{leaf}\Delta S_V - H_d}{T_{leaf}R}}}, \qquad (S25)$$

$$f_{J_{max}} = e^{\frac{H_{a\,J}(T_{leaf} - 298.15)}{298.15 T_{leaf} R}} \frac{1 + e^{\frac{298.15\Delta S_J - H_d}{298.15R}}}{1 + e^{\frac{T_{leaf}\Delta S_J - H_d}{T_{leaf}R}}}, \qquad (S26)$$

$$\Delta S_V = 668.39 - 1.07T_{air}, \qquad (S27)$$

$$\Delta S_J = 659.7 - 0.75T_{air}, \qquad (S28)$$

$$f_R = e^{0.1012(T_{leaf} - 298.15) - 0.0005\left((T_{leaf} - 273.15)^2 - 25^2\right)}, \qquad (S29)$$

where $H_{a\,V}$ is 72000 J/mol, $H_{a\,J}$ is 50000 J/mol, R is 8.314 J/mol/K, H_d is 200000 J/mol, T_{air} (°C) is the growth air temperature, T_{leaf} (K) is the leaf temperature.

The formula of the P-model

The detailed derivation process and parameter meanings can be obtained from the original articles of the P-model (Wang et al. 2017, 2023, Smith et al. 2019). The result of P-model is:

$$m = \frac{C_{ch} - \Gamma^*}{C_{ch} + 2\Gamma^*}, \tag{S30}$$

$$\omega = -(1 - 2\theta) + \sqrt{(1 - \theta)\left(\frac{1}{\frac{4c}{m}\left(1 - \theta\frac{4c}{m}\right)} - 4\theta\right)}, \tag{S31}$$

$$\omega^* = 1 + \omega - \sqrt{(1 + \omega)^2 - 4\theta\omega}, \tag{S32}$$

$$V_{c\,max} = \varphi I \frac{C_{ch} + K}{C_{ch} - 2\Gamma^*}\frac{\omega^*}{8\theta}, \tag{S33}$$

$$V_{c\,max,25} = \frac{V_{c\,max}}{f_{V_{c\,max}}}, \tag{S34}$$

$$T = \frac{LMA(C_{ch} - 2\Gamma^*)}{30 \times 0.0864\varphi I \frac{\omega^*}{8\theta}}\sqrt{\frac{2 \times 768 \times 13.23 f_{V_{c\,max}}}{(C_{ch} + K)(C_{ch} + \Gamma^*)}}, \tag{S35}$$

where φ is 0.257 and θ is 0.85. c is the cost parameter of the following assumption:

$$\frac{\partial A_j}{\partial J_{max}} = c. \tag{S36}$$

c is obtained through parameter tuning. The value of c in the study of Wang et al. (Wang et al. 2017) is 0.103 and the value of c in the study of Smith et al. (Smith et al. 2019) is 0.053.

Reference

Ball JT, Woodrow IE, Berry JA (1987) A Model Predicting Stomatal Conductance and its Contribution to the Control of Photosynthesis under Different Environmental Conditions. In: Biggins J (ed) Progress in Photosynthesis Research. Springer Netherlands, Dordrecht, pp 221–224. http://link.springer.com/10.1007/978-94-017-0519-6_48 (3 September 2023, date last accessed).

Bernacchi CJ, Pimentel C, Long SP (2003) *In vivo* temperature response functions of parameters required to model RuBP-limited photosynthesis: Modelling RuBP-limited photosynthesis. Plant, Cell & Environment 26:1419–1430.

Buckley T, Cescatti A, Farquhar G (2013) What does optimization theory actually predict about crown profiles of photosynthetic capacity when models incorporate greater realism? Plant Cell & Environment 36:1547–1563.

Farquhar G, Caemmerer S, Berry J (1980) A biochemical-model of photosynthetic co2 assimilation in leaves of c-3 species. PLANTA 149:78–90.

Heskel MA, O'Sullivan OS, Reich PB, Tjoelker MG, Weerasinghe LK, Penillard A, Egerton JJG, Creek D, Bloomfield KJ, Xiang J, Sinca F, Stangl ZR, Martinez-de la Torre A, Griffin KL, Huntingford C, Hurry V, Meir P, Turnbull MH, Atkin OK (2016) Convergence in the temperature response of leaf respiration across biomes and plant functional types. Proc Natl Acad Sci USA 113:3832–3837.

Kattge J, Knorr W (2007) Temperature acclimation in a biochemical model of photosynthesis: a reanalysis of data from 36 species. Plant, Cell & Environment 30:1176–1190.

Laĭsk AK, Nedbal L, Govindjee (eds) (2009) Photosynthesis in silico: understanding complexity from molecules to ecosystems. Springer, Dordrecht, Netherlands ; [New York].

Leuning R (1995) A critical appraisal of a combined stomatal-photosynthesis model for C3 plants. Plant Cell Environ 18:339–355.

Li Yong, Peng Shaobing, Huang Jianliang, Xiong Dongliang, Liu Xi (2013) Components and Magnitude of Mesophyll Conductance and Its Responses to Environmental Variations. Plant Physiology Journal 49:1143–1154.

Medlyn BE, Duursma RA, Eamus D, Ellsworth DS, Prentice IC, Barton CVM, Crous KY, De Angelis P, Freeman M, Wingate L (2011) Reconciling the optimal and empirical approaches to modelling stomatal conductance. Global Change Biology 17:2134–2144.

Prentice IC, Dong N, Gleason SM, Maire V, Wright IJ (2014) Balancing the costs of carbon gain and water transport: testing a new theoretical framework for plant functional ecology. Ecol Lett 17:82–91.

Smith NG, Keenan TF, Colin Prentice I, Wang H, Wright IJ, Niinemets Ü, Crous KY, Domingues TF, Guerrieri R, Yoko Ishida F, Kattge J, Kruger EL, Maire V, Rogers A, Serbin SP, Tarvainen L, Togashi HF, Townsend PA, Wang M, Weerasinghe LK, Zhou S (2019) Global photosynthetic capacity is optimized to the environment. Ecology Letters 22:506–517.

Wang H, Prentice IC, Keenan TF, Davis TW, Wright IJ, Cornwell WK, Evans BJ, Peng C (2017) Towards a universal model for carbon dioxide uptake by plants. Nature Plants 3:734–741.

Wang H, Prentice IC, Wright IJ, Warton DI, Qiao S, Xu X, Zhou J, Kikuzawa K, Stenseth NChr (2023) Leaf economics fundamentals explained by optimality principles. Sci Adv 9:eadd5667.

Xiong D, Douthe C, Flexas J (2018) Differential coordination of stomatal conductance, mesophyll conductance, and leaf hydraulic conductance in response to changing light across species. PLANT CELL AND ENVIRONMENT 41:436–450.
